# Flexible Polypyrrole‐Based pH Sensors via Oxidative Chemical Vapor Deposition

**DOI:** 10.1002/adhm.202505807

**Published:** 2026-02-03

**Authors:** Adrivit Mukherjee, Federico Ferrari, David Garcia Romero, Ilaria Squillante, Job Schoenmaker, Hamoon Hemmatpour, Anton Terpstra, Peter Dijkstra, Julien Es Sayed, L. Jan Anton Koster, Maria Antonietta Loi, Petra Rudolf, Giuseppe Portale, Ajay Giri Prakash Kottapalli, Marleen Kamperman, Ranjita K. Bose

**Affiliations:** ^1^ Chemical Product Engineering Faculty of Science and Engineering Engineering and Technology Institute Groningen (ENTEG) University of Groningen Groningen The Netherlands; ^2^ Polymer Science Faculty of Science and Engineering Zernike Institute for Advanced Materials (ZIAM) University of Groningen Groningen The Netherlands; ^3^ Bioinspired MEMS and Biomedical Devices Group Faculty of Science and Engineering Engineering and Technology Institute Groningen (ENTEG) University of Groningen Groningen The Netherlands; ^4^ Photophysics and Optoelectronics Faculty of Science and Engineering Zernike Institute for Advanced Materials (ZIAM) University of Groningen Groningen The Netherlands; ^5^ Physical Chemistry of Polymeric and Nanostructured Materials Faculty of Science and Engineering Zernike Institute for Advanced Materials (ZIAM) University of Groningen Groningen The Netherlands; ^6^ Surfaces and Thin Films Faculty of Science and Engineering Zernike Institute for Advanced Materials (ZIAM) University of Groningen Groningen The Netherlands

**Keywords:** bioelectronic sensors, electrically conductive polypyrrole, oxidative chemical vapor deposition, pH sensor, smart skin‐integrated wearables, spatiotemporal (bio)sensing, wound healing monitoring

## Abstract

The dynamic physicochemical environment of healing wounds provides valuable diagnostic information, with pH serving as a key biomarker for infection, inflammation, and tissue regeneration. However, the development of flexible, biocompatible, and stable pH sensors that can be seamlessly integrated into wearable platforms remains challenging. Here, we report a strategy to fabricate electrically conductive, pH‐responsive bioelectronic sensors based on ultrathin polypyrrole (PPy) films deposited via oxidative chemical vapor deposition (oCVD). The resulting flexible sensors enable monitoring of physiologically relevant pH changes (4–9) and exhibit modulation of electrical conductivity up to two orders of magnitude, reaching 304 S.cm^−1^ (pH 4). Grazing‐incidence wide‐angle X‐ray scattering reveals enhanced structural order and efficient *π–π* stacking with increasing dopant concentration, leading to improved charge transport. Complementary spectroscopic analyses demonstrate that reversible protonation‐deprotonation of the PPy backbone, governed by dopant counterion exchange, underlies the pH‐dependent electrical response. The all‐polymer pH sensors display high sensitivity, stability, and repeatability. Moreover, the substrate‐independent nature of oCVD enables the fabrication of pH‐sensing patches and spatially patterned micro‐islands, facilitating seamless integration into smart wound dressings for spatiotemporally resolved bioelectronic monitoring. This work advances the design of flexible, wearable pH sensors and provides opportunities for real‐time wound‐healing monitoring.

## Graphical abstract

Oxidative chemical vapor deposition (oCVD) of polypyrrole (PPy) thin films yields flexible, electrically conductive, and biocompatible pH sensors for monitoring on‐skin biological events. The highly sensitive, oCVD PPy skin‐conformal sensors enable real‐time, spatially resolved sensing of dynamic pH changes within physiologically relevant ranges (4–9) via reversible protonation‐deprotonation, advancing the fabrication of soft bioelectronic sensing interfaces for monitoring wound healing.

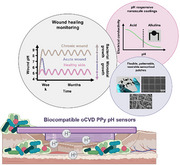

## 1 Introduction

Smart wearable technology has revolutionized personalized healthcare by addressing the limitations of traditional, bulky, rigid biosensing and treatment devices. This rigidity has hindered the advancement of next‐generation personalized medical devices, which emphasize real‐time health monitoring and tailored treatment feedback. The emergence of wearable, flexible on‐skin sensors offers a promising solution, enabling continuous monitoring of the physiological health status of biological events such as wound healing through minimally invasive means [1]. Bioelectronic sensors play a crucial role in early disease detection and proactive intervention, significantly enhancing patient outcomes [2, 3]. They are primarily designed to detect and monitor physical, chemical, electrical, and biological signals essential for drug delivery and wound theranostics, offering on‐demand therapy to enhance wound healing [4, 5, 6, 7, 8]. Monitoring physiological parameters, such as oxygenation, pressure, and moisture, in the dynamic wound environment presents significant challenges yet offers essential insights into its status [9]. Physicochemical (bio)markers, including uric acid (UA), glucose, pH, and immune proteins, are also modulated during the healing process of a wound [10, 11].

While many biosensors have focused on amperometric UA and oxygen detection, pH sensing in wound exudates has recently garnered considerable attention as a (bio)marker due to its dynamic changes during wound healing [12, 13, 14, 15, 16]. Typically, the human body maintains a neutral pH of 7.4, but healthy skin has an “acidic mantle” (pH 4‐6), as termed by Schad [17], regulated by enzymes and cellular activities [18, 19]. However, the integrity of this acidic milieu is compromised in wounds, allowing higher pH body fluids to breach the affected area. The pH of a wound can vary, with different values indicative of various healing stages [10]. For instance, wound repair mechanisms operate in a slightly acidic environment, with pH levels around 5‐6 [9]. Chronic wounds tend to have higher pH levels, sometimes rising to pH 10 due to bacterial proliferation. A positive relationship between pH levels and wound healing suggests that decreasing pH levels from alkaline to neutral indicates progression toward wound healing. A slightly acidic environment fosters fibroblast proliferation, promotes epithelization and angiogenesis, controls bacterial colonization, and facilitates oxygen release [9, 20]. Therefore, significant differences in wound pH can provide an early alert of infection, making pH a key (bio)indicator of the wound healing progress [21, 22]. Excluding pH data hampers dynamic wound monitoring and can lead to inaccuracies in multimodal sensing, such as estimates of UA concentration, due to pH‐dependent enzyme activity [12]. Thus, even slight pH changes can significantly impact the wound healing process [9, 23, 24].

Various (bio)sensor technologies, from color‐changing pH‐sensitive films to electrochemical pH systems, face significant challenges such as dye leaching and biofouling of electrodes, leading to reduced functionalities [25, 26, 27]. Recent progress has seen the development of temporary tattoo pH sensors for continuous skin monitoring, emphasizing the need for conformal attachment to ensure unhindered movement [28]. However, most of the reported examples of the sensors are predominantly colorimetric, necessitating subjective inspection, or electrochemical, measuring H^+^ ion concentration, with additional examples including protein sensors [29, 30]. Advances in flexible electronics and biomaterials have enabled the development of wearable epidermal biosensors, but they still encounter issues like sensor degradation and electrode fouling from wound exudate and sweat, indicating that exploring low surface energy sensing elements could offer potential solutions [31, 32]. Furthermore, the integration of electrochemical systems with electronic circuitry and power sources for data analysis remains a challenge [1, 2]. Developing diagnostic flexible electronics presents challenges in establishing a theranostic system within wound bandages [32]. This field is currently dominated by vapor phase deposition of carbonaceous and inorganic materials [33, 34]. Furthermore, advanced electrode arrays have been developed using laser‐induced graphene (LIG) via laser irradiation technology [35, 36, 37].

Electrically conductive polymers, including polypyrrole (PPy), polyaniline (PANi), and poly(3,4‐ethylenedioxythiophene) (PEDOT), have emerged as promising materials for a wide range of biomedical applications such as biosensing, drug delivery, and tissue engineering [38, 39, 40]. Among these, PPy stands out due to its ability to undergo a reversible doping and dedoping process involving its pyrrole nitrogen atoms and, therefore, is utilized in several reported applications [41, 42, 43]. This process is characterized by the oxidation and reduction of the nitrogen in the pyrrole ring, where the oxidized state (PPy^+^) corresponds to the conductive form, and the reduced state (PPy) corresponds to the non‐conductive form. Upon oxidation, PPy forms polarons (radical cations) and bipolarons (dications), which play pivotal roles in modulating the overall electrical conductivity [44]. The formation of polarons and bipolarons within the PPy backbone creates localized states in the band gap, and the excess charges enhance the electrical conductivity. This reversible transition between different states of PPy results in significant changes in its functional properties. For instance, the electrical resistance of PPy has been effectively leveraged in biosensing applications, while its volume expansion properties have found utility in drug delivery systems [12, 45].

In this work, we present a fabrication strategy for developing soft and flexible oCVD PPy‐based electrically conductive pH sensing patches as bioelectronic sensors for wound healing monitoring. Our fabrication process yields ultrathin, environmentally friendly conductive sensors, harnessing the pH responsiveness of oCVD PPy. Through comprehensive spectroscopic analyses including UV‐Vis‐NIR, FTIR, and XPS, we investigated the mechanistic changes in the oCVD PPy polymer structure due to counterion exchange, revealing that its electrical conductivity can be tuned up to 304 S.cm^−1^ by equilibrating the films in low pH solutions. GIWAXS studies were performed to reveal the structural ordering in the oCVD PPy chains with respect to dopant concentration. We demonstrate that the electrically conductive oCVD PPy films enable linear pH sensing in the physiologically relevant range of 4–9, demonstrating high sensitivity and repeatability for real‐life scenarios, such as acute and chronic wound healing processes. Furthermore, the ultrathin oCVD PPy can be conformally coated on soft, flexible, and porous electrospun polyacrylonitrile (PAN) fiber mats, fabricating smart pH‐responsive 3D patches with potential applications in (bio)sensing, such as monitoring the wound environment during healing. We also illustrate that the highly pH‐sensitive oCVD PPy can be spatially patterned into electrically conductive microdomains on various substrates, including polycarbonate sheets, paper, and disposable temporary tattoos.

## 2 Results and Discussion

### 2.1 pH Responsiveness of oCVD PPy

**FIGURE 1:**
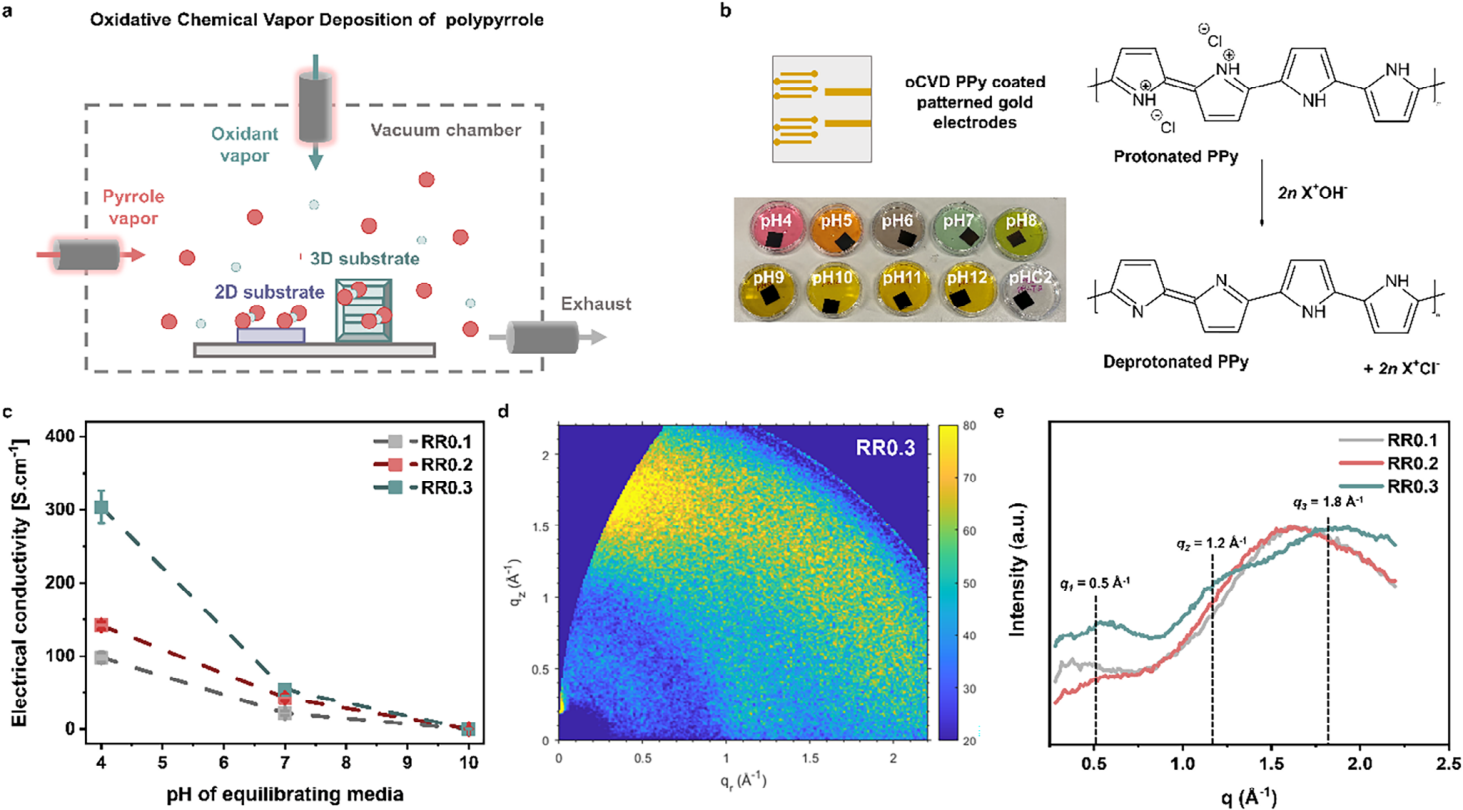
pH Sensitivity of the Electrical Conductivity of oCVD Polypyrrole. (a) Schematic of the oxidative chemical vapor deposition (oCVD) reaction setup used for conformal deposition of electrically conductive polypyrrole (PPy) onto diverse 2D substrates such as silicon wafer, glass substrate with patterned gold electrodes, Teflon sheet, and paper, as well as 3D substrates such as electrospun nanofiber mats. (b) Schematic of oCVD PPy‐coated (≈ 100 nm) glass substrates with patterned gold electrodes used for electrical measurements. Below, the photographs show oCVD PPy films equilibrating in buffer solutions ranging from pH 4‐12 (sample C2 refers to artificial wound exudate maintained at pH 7.2). Chemical structures representing the protonated and deprotonated states of oCVD PPy. (c) Plot of the electrical conductivity of oCVD PPy as a function of the pH of the equilibrating media, measured for different reactant ratios of oCVD PPy (RR0.1, RR0.2, and RR0.3). The conductivity decreases with increasing pH, showing a significant drop around neutral pH, and stabilizes in more alkaline (>pH 10) conditions. Furthermore, oCVD PPy chains with an initially higher dopant concentration (RR0.3) exhibit higher sensitivity to changes in electrical conductivity with increasing pH. Error bars represent standard deviation (*n* = 4). (d) 2D GIWXAS pattern of oCVD PPy films with high dopant concentration (RR0.3) showing anisotropic scattering, (e) Radially averaged scattering intensity plot of GIWAXS, illustrating that a higher dopant concentration promotes tighter interchain packing and orientation of the electrically conductive oCVD PPy polymer chains.

The PPy employed in this study was synthesized using the oCVD technique, as shown schematically in Figure 1a. Operated under mild conditions, the detailed experimental procedure is summarized in Section 4.2, resulting in doped oCVD PPy thin films with electrical conductivities on the order of 10^2^ S.cm^−1^ [46, 47]. Ultrathin coatings of oCVD PPy (thickness ≈ 100 nm) were uniformly deposited onto glass substrates patterned with gold electrodes. In a prior study, Dianatdar et al. reported that due to the in situ doping step during oCVD, increasing the oxidant‐to‐monomer ratio (referred to as the reactant ratio, RR) in the gaseous reactant feed resulted in chlorine‐doped PPy chains with higher dopant concentrations [48]. Given the significant influence of doping concentration on the electrical properties of conductive polymers, we prepared oCVD PPy films with varying reactant ratios (RR0.1, 0.2, and 0.3) for our preliminary investigation (Figure 1a) [49, 50, 51]. The deposition time for the oCVD process was adjusted to deposit PPy films with varying reactant ratios RR0.1, 0.2, and 0.3 but comparable film thicknesses (≈100 nm) onto the gold electrodes.

The in‐plane electrical conductivity of the oCVD PPy films was investigated as a function of increasing pH. Investigation into the in‐plane electrical conductivity of the oCVD PPy films involved equilibration in solutions spanning a pH range from 4 to 12, as depicted in Figure 1b, and thereafter their electrical conductivity was measured using a four‐point probe method. Figure 1c illustrates the electrical conductivity of the oCVD PPy as a function of increasing pH. The oCVD PPy equilibrated in acidic solutions exhibited electrical conductivities as high as 304 S.cm^−1^ (RR0.3), one of the highest electrical conductivities reported in the literature for oCVD PPy thin films [46, 52, 53]. As shown, the electrical conductivity of the oCVD PPy films equilibrated in pH 4 solutions demonstrated higher electrical conductivities with increasing RR, which could be attributed to the initially higher (oxidative) dopant concentration in the films and is investigated in detail later [49, 54]. However, regardless of the dopant (RR) concentration, all oCVD PPy samples exhibited a significant decrease in electrical conductivity as pH increased from 4 to 10, indicating a strong dependence on the pH of the equilibrating medium, possibly due to counterion exchange [55]. For instance, the electrical conductivity of the acid‐doped RR0.3 oCVD PPy film, initially measured at 304 ± 22 S.cm^−1^ at pH 4, decreased by approximately fivefold to 54 ± 7.8 S.cm^−1^ under neutral conditions (pH 7), and further dropped drastically to 0.31 ± 0.03 S.cm^−1^ when equilibrated in alkaline conditions (pH 10). This indicates that the acid‐doped oCVD PPy chains could be stabilized in their protonated state and therefore show high electrical conductivity. The results suggest that in acidic conditions, the oxidized oCVD PPy chains are protonated with H^+^ ions and doped with Cl^−^ counterions, resulting in a highly conductive state. In contrast, under alkaline conditions, the conductive oxidized PPy chains likely undergo deprotonation by OH^−^ counterions, leading to neutralization of the backbone chains and a significantly less conductive state (see Figure 1b) [12, 49]. Similarly, the electrical conductivity of RR0.2 and RR0.1 oCVD PPy films decreased from 140 ± 5.3 and 98 ± 7.1 S.cm^−1^ at pH 4, to 43 ± 4.1 and 22 ± 3.0 S.cm^−1^ at pH 7, respectively, with further reductions observed at higher pH. This suggests that the higher initial doping (achieved in this study with increasing RR) of the oCVD PPy chains results in a larger decrease in electrical conductivity. Moreover, all the oCVD PPy films, irrespective of their initial doping concentrations, saturate to similar electrical conductivities at very high pH. These results also indicate that the availability of the dopant counterion governs the mechanism for the change in electrical conductivity of oCVD PPy. Therefore, leveraging the decrease in electrical conductivity in the physiologically relevant range of 4–9, we hypothesized that oCVD PPy could be utilized in pH sensing applications such as wound healing monitoring due to changes in H^+^ ions [12, 50].

Given the significant influence of the initial doping concentration on the electrical conductivity of oCVD PPy films, grazing incidence wide‐angle X‐ray scattering (GIWAXS) measurements were performed to examine the interchain packing and orientation of PPy chains at increasing dopant concentrations, as indicated by the reactant ratio (RR = [oxidant]/[monomer]) during oCVD. The 2D GIWAXS pattern (plotted as a function of the parallel *q_r_* vs. quasi‐vertical *q_z_* scattering vectors) of oCVD PPy films with RR = 0.3 is shown in Figure 1d, while the ones with RR = 0.2 and RR = 0.1 are reported in Figures S1a,b. The GIWAXS pattern for the oCVD PPy film with RR = 0.3 clearly exhibits anisotropic scattering, indicating a preferential orientation of the oCVD PPy crystallite domains. The most dominant signal is located at *q* ≈ 1.8 Å^−1^, which corresponds to an interplanar distance of 3.48 Å in agreement with the distance expected for a high‐quality *π–π* stacking system [56, 57, 58]. The majority of this scattering intensity is concentrated along the vertical *q_z_* (*q_y_* = 0) direction, indicating a dominant face‐on orientation of oCVD PPy chains on the substrate. A similar signal is also observed for the films with lower RR, but with a much weaker signal anisotropy (Figure S1a,b), meaning that the crystallites are more randomly oriented. Figure 1e presents the radially averaged total scattered intensity as a function of the scattering vector, *q*. All data have been background‐subtracted and normalized for film thickness. The oCVD PPy film with RR = 0.3 exhibits three clear peaks located at 0.5 Å^−1^ (*d*‐spacing = 12.6 Å), 1.2 Å^−1^ (*d*‐spacing = 5.2 Å), and at the above‐mentioned 1.8 Å^−1^ position (*d*‐spacing = 3.48 Å). We can associate these distances with the dimensions of an approximated crystalline unit cell of the oCVD PPy, corresponding to the [001] (*c*‐axis), [100] (*a*‐axis), and [010] (*b*‐axis), respectively. Interestingly, for the lowest RRs, the [100] and [001] peaks are not clearly visible, and the [010] peak is sensibly lower and shifted to lower *q*‐values with respect to the RR = 0.3 film, indicating a more disordered packing and a significantly lower *π–π* stacking. This trend, together with the recorded higher anisotropy of the [010] peak for the RR = 0.3 film, reveals that a higher dopant concentration promotes a stronger preference for oCVD PPy chain to orient face‐on with the substrate and a significantly enhanced *π–π* orbital overlap. To better quantify the difference in crystallite orientation between the RR = 0.3 and the RR = 0.1 and 0.2 films, intensity profiles along the horizontal (*q_y_*) and quasi‐vertical (*q_z_*) directions have been computed, together with the azimuthal distribution of the [010] peak (Figure S1d–f). In fact, in addition to the preferred orientation of oCVD PPy chains, the degree and amount of orientation significantly influence the charge transport properties of semiconducting films, ultimately impacting electrical conductivity [58]. The azimuthal intensity profiles of oCVD PPy films with varying dopant concentrations (shown in Figure S1f) reveal that higher doping concentrations (RR = 0.3) promote a significantly higher degree of orientation compared to films with lower dopant concentrations, which exhibit a lower orientation amount. Therefore, collectively the GIWAXS results indicate that increasing the dopant concentration in oCVD PPy films enhances structural ordering and face‐on crystallite orientation, promoting a more compact *π–π* stacking, which in turn improves carrier transport properties and leads to significantly higher electrical conductivity as shown in Figure 1c.

### 2.2 Mechanism of pH Response of oCVD PPy

To explore the underlying mechanism behind the modulation of functional electrical conductivity in oCVD PPy chains in response to pH changes in the equilibrating media (Figure 1c), electronic band transitions within oCVD PPy films were examined using UV‐vis‐NIR spectroscopy. The absorption spectra of the oCVD PPy films (RR0.2, thickness ≈ 100 nm) were recorded in the range of 350‐2000 nm after equilibrating in solutions with varying pH from 4 to 12.

**FIGURE 2:**
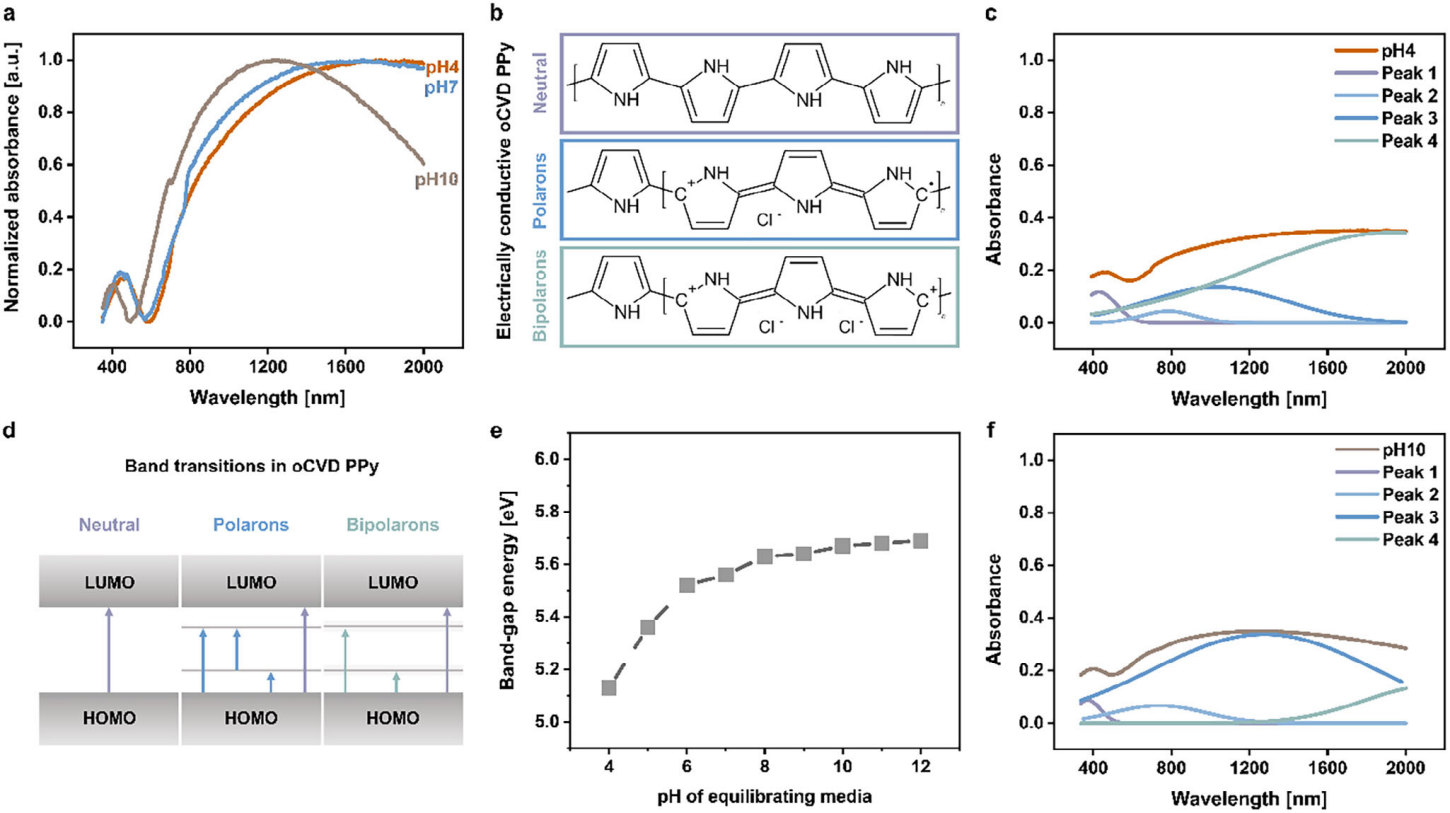
Influence of pH on the Electronic Band Transitions of oCVD Polypyrrole. (a) Normalized absorbance spectra of oCVD PPy equilibrated at pH 4, pH 7, and pH 10, demonstrating significant changes in optical properties across the UV‐vis‐NIR spectrum as a function of pH. (b) Chemical structures representing oCVD PPy in its neutral, polaron, and bipolaron states. (c) Deconvolution of the absorbance spectrum of oCVD PPy films equilibrated to pH 4 into four distinct peaks, illustrating the contributions of various electronic transitions to the overall absorbance. The deconvolution of the absorbance spectrum of PPy at pH 10 (f) shows the altered individual peak contributions compared to acidic conditions. (d) Schematic energy band diagrams corresponding to neutral chains, polarons, and bipolarons in PPy. These diagrams depict the electronic transitions from the HOMO to the LUMO, including intermediate states associated with polarons and bipolarons. (e) Plot of the band‐gap energy of oCVD PPy as a function of the pH of the equilibrating medium.

Figure 2a shows the normalized representative absorbance spectra of oCVD PPy equilibrated to pH 4, 7, and 10. The absorbance spectra of oCVD PPy films equilibrated at pH 4 exhibit a distinct absorption maximum at 477 nm (2.60 eV), characteristic of the interband transitions of the neutral oCVD PPy chains, and a broad peak in the lower energy regime indicative of cumulative contributions from allowed optical band transitions below the interband transitions [44, 49, 59, 60]. This suggests an inherently doped structure of the oCVD PPy chains. Kaufmann et al. summarized that in non‐degenerate ground‐state polymers such as PPy, introducing defects, such as removing a single electron from the PPy backbone through doping, accompanied by lattice relaxation, generates a polaron that allows multiple optical transitions below the interband transition, as this is energetically favorable [44]. Due to the non‐degeneracy of the ground state, further doping of the PPy chains and subsequent electron removal either leads to the generation of more polarons or to the interaction and rearrangement of free polarons to form bipolarons, at a rate characteristic of ionic diffusion that can make further optical transitions possible [44]. Optical studies into the electrical conduction mechanism of doped PPy chains by Yakushi et al. concluded the coexistence of neutral conjugated segments alongside polaron and bipolaron states in doped PPy chains (shown schematically in Figure 2b) [61]. Therefore, to further investigate the contributions of individual band transitions in the heterogeneous doped oCVD PPy system, the absorbance spectra of the oCVD PPy films equilibrated in solutions with different pH (4‐12) were deconvoluted using Gaussian functions.

The deconvoluted absorbance spectra of the oCVD PPy films equilibrated in acidic (pH 4) and alkaline (pH 10) conditions are shown in Figure 2c,f, respectively. These spectra exhibit four characteristic peaks corresponding to the transitions (shown schematically in Figure 2d): peak 1 at the highest energy (2.6 eV) corresponds to the interband transition of the neutral chains; peak 2 at 1.6 eV can be attributed to the transition from a lower polaron level to a higher polaron level or from the HOMO to the upper polaron band; peak 3 at 1.2 eV can be attributed to the excitation from the HOMO level to the lower polaron band; and peak 4 at 0.64 eV corresponds to excitation from the HOMO level to the lower bipolaron bands [44, 54]. The deconvoluted spectra of all other samples are shown in Figure S2. Interestingly, as the pH of the equilibrating media increases, the absorbance spectra of the oCVD PPy films undergo a distinct blue shift (blue shift of peak 1 attributed to interband transitions of the neutral oCVD PPy chains at 2.6 eV for pH4 to 3.3 eV for pH10), indicating the dominance of the electronic band transitions that require higher energy. This could imply that the minimum energy required for an electron to undergo an interband transition increases, which can be attributed as a natural consequence of the deprotonation of the oCVD PPy chains [54, 62, 63]. Furthermore, peaks 2–4, related to the polaron and bipolaron transitions of the doped conjugated PPy chains, also undergo significant alterations after being equilibrated in solutions of different pHs. Figure 2c shows that the oCVD PPy films equilibrated in acidic pH 4 are mostly dominated by bipolarons (peak 4). As the pH of the equilibrating media is increased, the absorption bands related to the bipolarons grow weaker in amplitude while the polarons grow relatively stronger. This behavior changes as the absorbance spectra of the oCVD PPy films equilibrated in solutions of pH 8 and above are dominated by the peaks attributed to the polarons. Interestingly, while the absorbance peak related to the interband transitions of the neutral chains was blue‐shifted to higher energy, its relative intensity remained consistent with changes in the pH of the equilibrating media. Additionally, absorption curves reveal that all the states ‐ neutral, polarons, and bipolarons persist across all films, including the most oxidized (pH 4) and reduced (pH 12) states. This suggests the coexistence of pyrrole rings characteristic of both forms of the polymer, contributing to both polaron and bipolaron carriers and thus overall electrical conductivity, as hypothesized by Genoud et al. [64, 65] The data imply that the pH of the equilibration media, which governs the presence of doping counterions, modulates the presence of polarons and bipolarons contributing to the overall functional electrical conductivity of the oCVD PPy thin films.

These findings are consistent with those reported by Brédas et al., who found that the protonation of the conjugated polymer backbone influences the geometric modifications of the electronic structure such that, in comparison to the undoped case, two bipolaron states appear in the gap: the HOMO level is pushed up in energy, and the LUMO level is pushed down [66]. This explains the blue shift of the absorbance spectra of the oCVD PPy when equilibrated in solutions with increasing pH, leading to deprotonation of the oCVD PPy chains. Figure 2e illustrates the change in bandgap energy across oCVD PPy films equilibrated in pH 4 to 12 solutions, which was further confirmed using Tauc plots (Figure S3), showing a blueshift in absorption onset with increasing pH of the equilibrating media. Therefore, all the evidence together strongly suggests that at low pH, the doped oCVD PPy chains mainly exist in their protonated state with dominating bipolarons leading to their high electrical conductivity in the order of 10^2^ S.cm^−1^. As the pH of the equilibrating media increases, the oCVD PPy chains undergo deprotonation due to the extraction of the doping counterions, leading to a drastic decrease in the electrical conductivity in the range of 10^−1^ S.cm^−1^ [49, 67].

**FIGURE 3:**
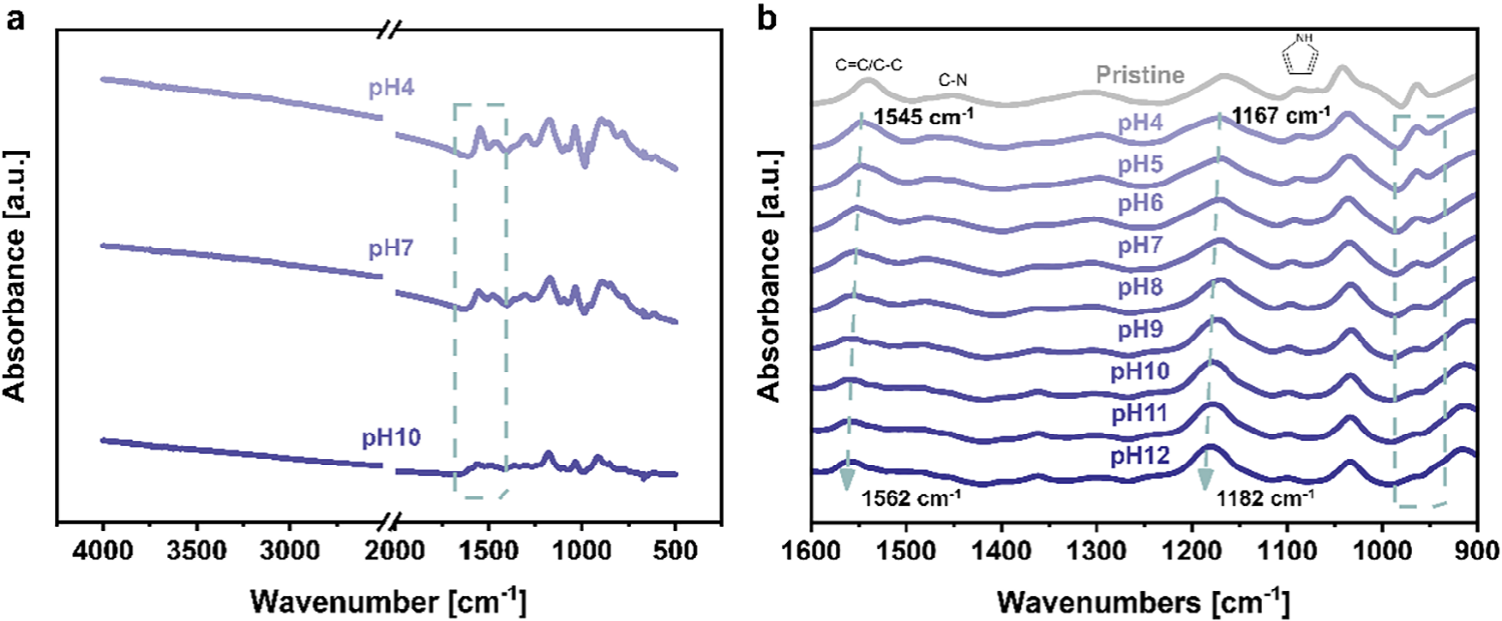
FTIR Spectra of oCVD Polypyrrole with Varying pH. (a) FTIR spectra of oCVD PPy equilibrated at pH 4, pH 7, and pH 10, demonstrating the changes in absorbance with varying pH, (b) Fingerprint region of the FTIR spectra of PPy equilibrated at different pH levels (pH 4‐12) and compared to pristine PPy as a control. The absorption bands corresponding to the characteristic peaks of oCVD PPy: *ν_C = C/C‐C_* (1545 cm^−1^) and *ν_C‐N_* (1454 cm^−1^) are highlighted. The changes in these bands reflect the protonation and deprotonation processes affecting the structure of the polymer backbone as the pH varies, which can be correlated with the observed shifts in the electronic and optical properties.

Figure 3 shows the FTIR absorbance spectra of the oCVD PPy coatings equilibrated in solutions of pH 4, 7, and 10. The monotonic rise in absorbance in Figure 3a above 1600 cm^−1^ for the oxidized PPy film in its protonated state (pH 4) is attributed to vibrational transitions of the different oxidized and reduced species of oCVD PPy [68, 69]. This well‐known phenomenon for conductive polymers such as PPy indicates a doped backbone structure, which could explain the high electrical conductivity of oCVD PPy films achieved in this study when equilibrated in low pH solutions. Figure 3a illustrates a notable reduction in the broad absorption band of oCVD PPy films when equilibrated in solutions with higher pH values (7 and 10), indicating a shift towards dominance of free carrier excitations (intrachain), suggestive of deprotonation of the oCVD PPy chains [63]. This observation correlates with a significant decrease in the electrical conductivity of the films to the order of 10^−1^ S.cm^−1^. Additionally, in Figure 3a, the absorption bands within the fingerprint region (1600‐900 cm^−1^) of the FTIR spectra exhibit a stronger intensity in the oxidized oCVD PPy films compared to those equilibrated in solutions of higher pH.

The characteristic bands in the spectrum of the oCVD PPy at pH 4 are attributed to *ν_(C = C/C‐C)_* and *ν_b(C‐N)_*, located at 1545 cm^−1^ and 1454 cm^−1^, respectively (Figure 3b) [68]. In contrast, the spectra of the oCVD PPy films equilibrated at higher pH values exhibit a successive blue shift of the 1545 cm^−1^ peak with increasing pH, reaching 1562 cm^−1^ in the most reduced oCVD PPy film at pH 12. Furthermore, the 1454 cm^−1^ peak undergoes a blue shift to 1481 cm^−1^, accompanied by a significant reduction in intensity with increasing pH. This indicates a shorter delocalization length of charges along the poly‐conjugated oCVD PPy system, suggesting the dedoping of the oCVD PPy chains [70, 71]. This observation aligns with the decreased electrical conductivity observed in the films, as reported by earlier investigations. Additionally, the absorption bands attributed to *ν_b(pyrrole ring)_* at 1167 cm^−1^ in the pH 4 spectrum undergo a significant blue shift to 1182 cm^−1^, while the band at 964 cm^−1^ gradually diminishes, nearly disappearing in the pH 12 spectrum [72, 73]. The peak at 1091 cm^−1^ (pH 4) from *δ_(N‐H_^+^_)_* shifts slightly to 1098 cm^−1^ (pH 12) [73]. However, the peak positions of absorbance bands around 1302 cm^−1^ and 1035 cm^−1^ attributed to *δ_(C‐H/C‐N)_* and *δ_(C‐H/N‐H)_*, respectively, appear unaffected by the deprotonation process when equilibrated in higher pH solutions, as predicted by Tian and Zerbi [68, 69].

XPS was further utilized to investigate the deprotonation of PPy coatings following exposure to aqueous solutions with varying pH levels. The investigation involved analyzing the control sample before exposure and samples after exposure. As illustrated in Figure S4, the survey spectrum of the control sample revealed the presence of PPy constituent elements C and N, as well as Sb and Cl originating from the doping agent SbCl_5_. Additionally, a small signature of O was detected, which could be associated with the overoxidation of the PPy rings during the oxidative polymerization process [48]. Following exposure of the PPy coatings to equilibrating solutions, the signatures of Sb and Cl were observed to diminish significantly (Figure S4). The stoichiometric analysis, which involved collecting detailed core‐level spectra of C1*s*, N1*s*, O1*s*, Sb*3d*, and Cl*2p* for all samples and deducing the corresponding atomic percentages in the probed volume, is presented in Table 1. The atomic percentages of Sb and Cl exhibited a significant decrease in samples exposed to solutions with different pH levels. Specifically, the atomic percentage of Sb in the samples, which were exposed to solutions with varying pH levels, decreased from 8.8 at.% in the control sample to 0.1‐0.3 at.%. This reduction indicates that the impurities resulting from the doping agent (SbCl_5_) used during the oCVD process are prone to dissolution in the equilibrating media. Moreover, the atomic percentage of Cl decreased from 7.1 at.% in the control sample to 0.2–1.8 at.%, which is indicative of the deprotonation process that the coating undergoes when conditioned in solutions with pH values ranging from 5 to 11 [50]. These results align with the electrical conductivity of oCVD PPy films ‐ the electrical conductivity of the unexposed films (120 S.cm^−1^) increased to 140 S.cm^−1^ upon equilibration to pH 4, then decreased to 110 S.cm^−1^ at pH 5, and further reduced when equilibrated in solutions with increasing pH.

**TABLE 1:** Chemical composition of the surface of PPy coatings exposed to aqueous solutions with different pH levels.

PPy samples	Atomic percentage (± 2 at.%)				
	C	N	O	Sb	Cl
Untreated	64.6	12.7	6.8	8.8	7.1
pH 5	77.6	8.8	12.3	0.2	1.0
pH 8	76.7	11.1	10.1	0.3	1.8
pH 11	79.1	10.7	9.9	0.1	0.2

**FIGURE 4:**
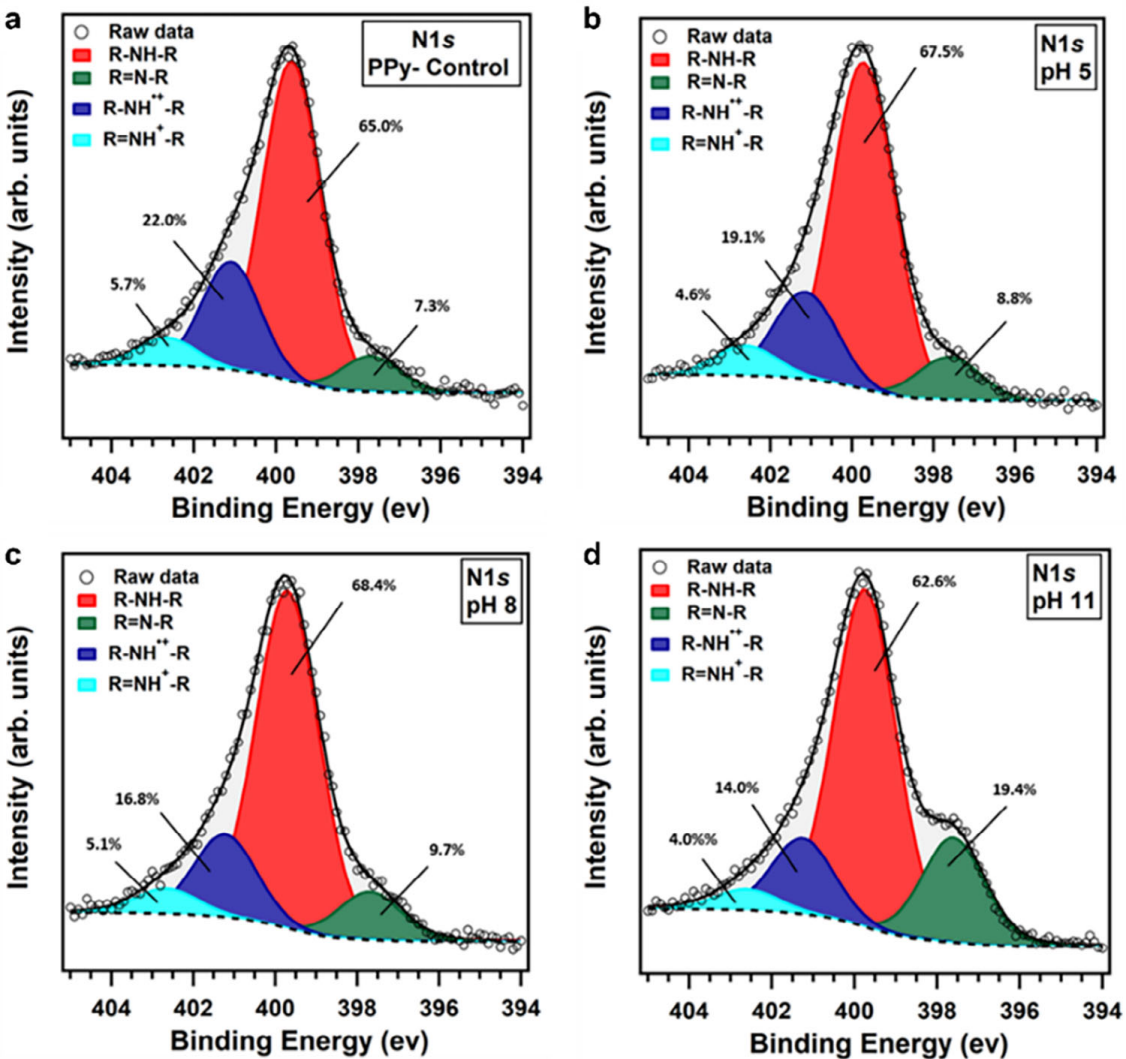
XPS spectra of the N1*s* core level region of PPy coatings after exposure to aqueous solutions with different pH levels.

Figure 4 shows the detailed XPS spectra of the N1*s* core level region; all spectra exhibit an asymmetrical peak that requires four components to obtain a good fit. The main peak located at a BE of 399.7 eV (indicated in red in Figure 4) is attributed to the ─NH─ species in the pyrrole ring, while the other components at a BE of 397.7 eV (marked in green), 401.2 eV (blue) and 402.6 eV (cyan) are attributed to C═N, C─N^+^, and C═N^+^ nitrogen species [48]. Figure 4 demonstrates that as the pH of the medium increases, the contribution of the peak associated with C═N nitrogen species becomes more pronounced, while the intensity of the components attributed to positively charged nitrogen decreases. Taken together, the relative percentage of positively charged nitrogen species (C─N^+^ and C═N^+^) decreases from 23.7% to 18%, and the relative percentage of C═N species increases from 8.8% to 19.4% as the pH of the medium is increased from 5 to 11, suggesting that the PPy coatings become more neutral when exposed to these environments. This is consistent with the pKa value of PPy, which ranges from 3 to 4 [50], indicating that the nitrogen species undergo deprotonation at pH values higher than the pKa of PPy.

Deconvolution of the C1*s* core level spectra of the PPy coatings shown in Figure S5 reveals that there are at least five distinct chemical species present. The main contribution to the C1*s* line, identified at a BE of 284.2 eV and marked in purple in Figure S5, is attributed to sp2 C═C species [48]. Additionally, three other components at BEs of 285.1 eV (indicated in red), 286.1 eV (indicated in green), and 287.3 eV (indicated in cyan) are assigned to C─N (pyrrolic N), C─O/C─N^+^/C═N, and C═O/C═N^+^ carbon species, respectively [74]. Another peak observed at a BE of 289.2 eV (indicated in orange) can be assigned to a shake‐up [74]. When the PPy coatings are exposed to solutions with pH values from 5 to 11, the relative intensity of the C─N component increases from 27.0% to 31.0%. This change is consistent with the interpretation of N1*s* core level spectra, where a more neutral PPy coating is expected to form in the media with higher pH values.

Figure S6 displays the deconvolution of the Cl*2p* core level spectra for the PPy coatings. As shown in Figure S6a, the Cl*2p* spectrum of the PPy‐control sample, before being exposed to the equilibrating solutions, requires three components for a satisfactory fit; the one at a BE of 197.4 eV (marked in yellow in Figure S6) is assigned to the Cl anion, the component peaked at a BE of 198.4 eV (marked in green) corresponds to Cl─N^+^ and the signal at a BE of 199.9 eV (marked in pink) is attributed to the covalently bonded Cl with carbon (C─Cl) [48]. The Cl─N^+^ species acts as an intermediary in the charge transfer process between the PPy backbone and chloride anions and ultimately contributes to the electrical conductivity of the coating [75]. In Figure S6b–d, it can be observed that the signal intensity assigned to this component significantly diminishes when the coating is exposed to solutions with pH values ranging from 5 to 11. This is due to the deprotonation process, resulting in the loss of HCl molecules from the coating, and correlates with the observed decrease in electrical conductivity (Figure 1c). Additionally, as the pH of the equilibration medium increases, there is a corresponding decrease in the atomic percentage of Cl anions. After exposure to a solution with pH 11, the Cl in the sample predominantly forms C─Cl bonds, suggesting that the charge of the oCVD PPy is compensated through the deprotonation process.

### 2.3 pH Sensing of oCVD PPy

**FIGURE 5:**
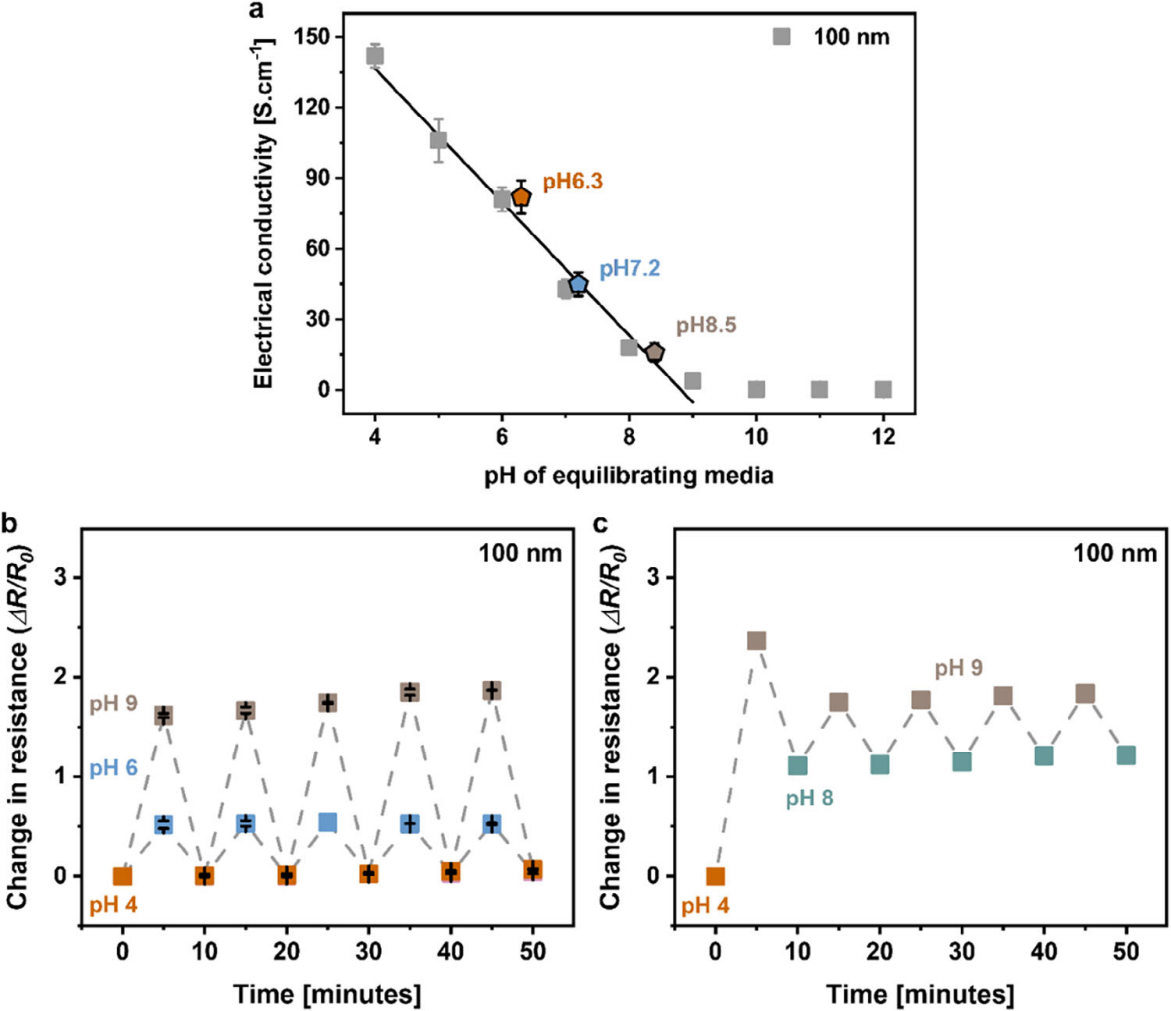
Demonstrating the Sensitivity of oCVD Polypyrrole for pH Sensing. (a) Electrical conductivity of oCVD PPy thin films (RR0.2, 100 nm) as a function of the pH of the equilibrating media. The conductivity decreases linearly with increasing pH due to deprotonation until pH 9 (*R^2^* = 0.99), after which it plateaus. These films were also used to measure the pH of artificial wound exudate maintained at specific pH levels (6.3, 7.2, and 8.5), demonstrating accurate pH measurement capabilities. Error bars represent the standard deviation (*n* = 4). (b) Relative change in resistance of oCVD PPy films during successive dynamic pH cycles (pH 4–6 and pH 4–9), indicating reversible behavior with some drift over time. This demonstrates the potential of PPy thin films for measuring dynamic pH changes. Error bars represent standard deviation (*n* = 3). (c) Demonstration experiment where the oCVD PPy film equilibrated at pH 4 was cycled between pH 8 and pH 9 to simulate the conditions of chronic wound healing, demonstrating the ability of oCVD PPy to sense dynamic changes in pH of the environment.

The electrical response of the oCVD PPy thin films to varying equilibrating pH levels (4‐10) is depicted in Figure 5. Figure 5a shows the in‐plane electrical conductivity of oCVD PPy thin films, derived from resistance values obtained using a four‐point probe method. To assess responsiveness and sensitivity, the samples were equilibrated in buffer solutions with incrementally increasing pH values.

At pH 4, the oCVD PPy film exhibited a conductivity of 140 ± 5.3 S.cm^−1^. As the pH increased, the conductivity decreased, dropping to 110 ± 9.2 S.cm^−1^ at pH 5 and further to 3.9 ± 0.5 S.cm^−1^ at pH 9. This decrease can be attributed to the reversible deprotonation due to the dopant counterion exchange of the oCVD PPy chains, as discussed in the earlier section. Beyond pH 10, the films lost most of their electrical conductivity, recording values below 1.0 S.cm^−1^, indicating the complete deprotonation of the PPy. Notably, the electrical conductivity of thicker (≈500 nm) oCVD PPy films (deposited according to oCVD PPy calibration curve shown in Figure S7) was also investigated using a similar protocol (equilibrated in buffer solutions for 5 min) to evaluate the effect of film thickness on the electrical response to pH changes. Figure S8 shows that while the thicker oCVD PPy films exhibited marginally higher electrical conductivity compared to the thin oCVD PPy films at alkaline pH values (10‐12), the thin oCVD PPy films were more sensitive to changes in pH in the range of pH 4–9, as indicated by the slope of the curve.

As discussed earlier, the change in electrical resistance of the oCVD PPy network is predominantly governed by the reversible protonation‐deprotonation process of the polymer backbone. In highly alkaline conditions, the electrical conductivity is primarily due to neutral and polaron domains, with conductivity increasing at higher pH levels as polarons rearrange to form bipolarons, a process kinetically limited by counterion diffusion [44]. Therefore, while the thicker oCVD PPy films exhibit higher inherent doping concentrations compared to the thinner films due to the in situ polymerization and doping achieved by the oCVD process, the protonation‐deprotonation process requires more equilibration time to reach saturation. In contrast, the thinner oCVD PPy films show lower response times to changes in pH, as the samples were equilibrated to saturation within 5 min and hence were further tested for applicability. In addition, oCVD PPy thin films were subjected to equilibration in artificial wound exudate with varying, predetermined pH levels (6.3, 7.2, and 8.5), followed by measurement of their electrical conductivities to evaluate the analytical reliability for real applications. The samples equilibrated at pH values of 6.3, 7.2, and 8.5 exhibited electrical conductivities of 96 ± 10, 45 ± 5.2, and 16 ± 5.2 S.cm^−1^, respectively. The results depicted in Figure 5a illustrate a linear correlation between the electrical response of the oCVD PPy and pH within the range of 4–9, suggesting applicability in accurately measuring the pH of biologically complex fluids such as wound exudates.

The resistance‐based pH sensing performance of oCVD PPy was further evaluated by assessing its ability to detect dynamic changes in the pH of equilibrating media within the range of 4–9. The oCVD PPy films (RR0.2, thickness ≈ 100 nm) were patterned using a shadow mask and deposited onto polycarbonate sheets. The electrical response to changes in resistance was measured over time using a custom‐built cell (described in Section 4.4 and shown in Figure S9). Initially, a calibration curve was plotted by measuring the electrical resistance of the oCVD PPy thin films equilibrated at pH 4, followed by subsequent changes in pH (Figure S8b). The electrical resistance of the oCVD PPy films demonstrated high sensitivity to changes in pH, with the resistance increasing by almost 60% when the pH increased from 4 to 6, and further by 168% when the pH was increased to 9. This calibration curve (Figure S8b) shows a linear regime of electrical resistance in the physiologically relevant range of pH 4 to 9, attributed to the reversible doping and dedoping process, making oCVD PPy a promising candidate for pH sensing applications.

Figure 5b shows the electrical response regarding the relative change in resistance of oCVD PPy films exposed to dynamic cycles of varying pH, demonstrating excellent sensitivity to both small and large changes in pH and showcasing a wide detection range of physiological relevance. The stability of the electrical response of the oCVD PPy pH sensing strip was tested by continuously measuring the electrical resistance while exposing it to solutions with pH values cycled between pH 4, 6, and 9. Figure 5b shows that the pH sensor exhibits reversible resistance changes in response to dynamically varying pH cycles over five consecutive cycles within an experimental duration of 50 min, with a marginal drift (≈ 5.2%) at the end of the test, indicating good cyclic stability and recovery of the fabricated pH sensor. To demonstrate the applicability of the fabricated oCVD PPy pH sensor in wound healing applications, a demonstration experiment was designed to simulate the conditions of a chronic wound healing process that involves sensing a large initial pH change (from pH 4 to pH 9 in this study) and thereafter sensing successive small dynamic changes in pH over longer periods (cycles between pH 9 and 8). Figure 5c demonstrates that the electrical response of the pH sensor can be directly correlated to the calibration curve (shown in Figure S8b), providing precise information on temporal dynamic changes in pH. The stability of the oCVD PPy‐based pH sensor was further evaluated through repeated bending tests. The relative changes in resistance of the oCVD PPy layer were recorded while the sensor strip was exposed to 300 bending cycles at bending strains of 0.5% and 1.0%. As shown in Figure S10, minor resistance variations are observed during the initial cycles, after which the sensor response stabilizes, exhibiting no further significant changes even after 300 cycles, with the total resistance drift remaining below 10% for both bending strains, confirming the good stability of the oCVD PPy sensing layer under repeated deformation. Conductive polymer‐based pH sensors have also been widely reported, using PANI and PEDOT derivatives. PANI‐based pH sensors typically rely on reversible protonation‐deprotonation of the emeraldine backbone and are commonly implemented as potentiometric devices, exhibiting near‐Nernstian sensitivities over a pH range of 5–8 [76, 77]. Nanostructured and composite PANI systems have even demonstrated super‐Nernstian responses up to ∼80 mV pH^−1^ over wide pH ranges (3–10) [78]. While these sensors often show fast response times, their long‐term operational stability can be compromised by dopant leaching, film delamination, and irreversible over‐oxidation under repeated pH cycling in aqueous environments. PEDOT‐based pH sensors, most commonly employing PEDOT:PSS, benefit from high intrinsic electrical conductivity and good electrochemical stability, which has led to their widespread use in bioelectronic devices [79]. However, the pH sensitivity of PEDOT‐based systems varies strongly with polymer formulation, post‐treatment, and device architecture, and reported responses are often low, particularly in alkaline conditions where polymer swelling and loss of charge carriers can occur, leading to a narrow detection range [80]. In comparison, the resistance‐based oCVD PPy sensors exhibit a linear, reversible electrical response within the physiologically relevant pH range of 4–9. Unlike many solution‐processed PANI and PEDOT systems, the vapor‐phase oCVD process yields ultrathin, conformal, and substrate‐independent PPy films with enhanced structural order and efficient *π–π* stacking, resulting in large conductivity modulation spanning nearly two orders of magnitude. Moreover, the oCVD PPy sensors demonstrate good repeatability under dynamic pH cycling, indicating improved operational stability compared to many previously reported conductive polymer‐based pH sensors [77, 80]. Therefore, the results collectively suggest that the oCVD PPy pH sensor simultaneously achieves high sensitivity, linearity, and a wide, physiologically relevant sensing range, making it well‐suited for potential biomedical applications such as wound healing monitoring.

### 2.4 Applicability to Advanced Fabrication Techniques for Flexible 3D Patches and Spatial Patterning

A key feature of wearable bioelectronic devices for wound healing monitoring is their ability to conform to the skin and maintain flexibility, which is essential for devices used in topical and transcutaneous diagnostics and therapeutics [2, 12]. Therefore, the applicability of oCVD to fabricate flexible conformal 3D pH‐sensing patches was evaluated.

**FIGURE 6:**
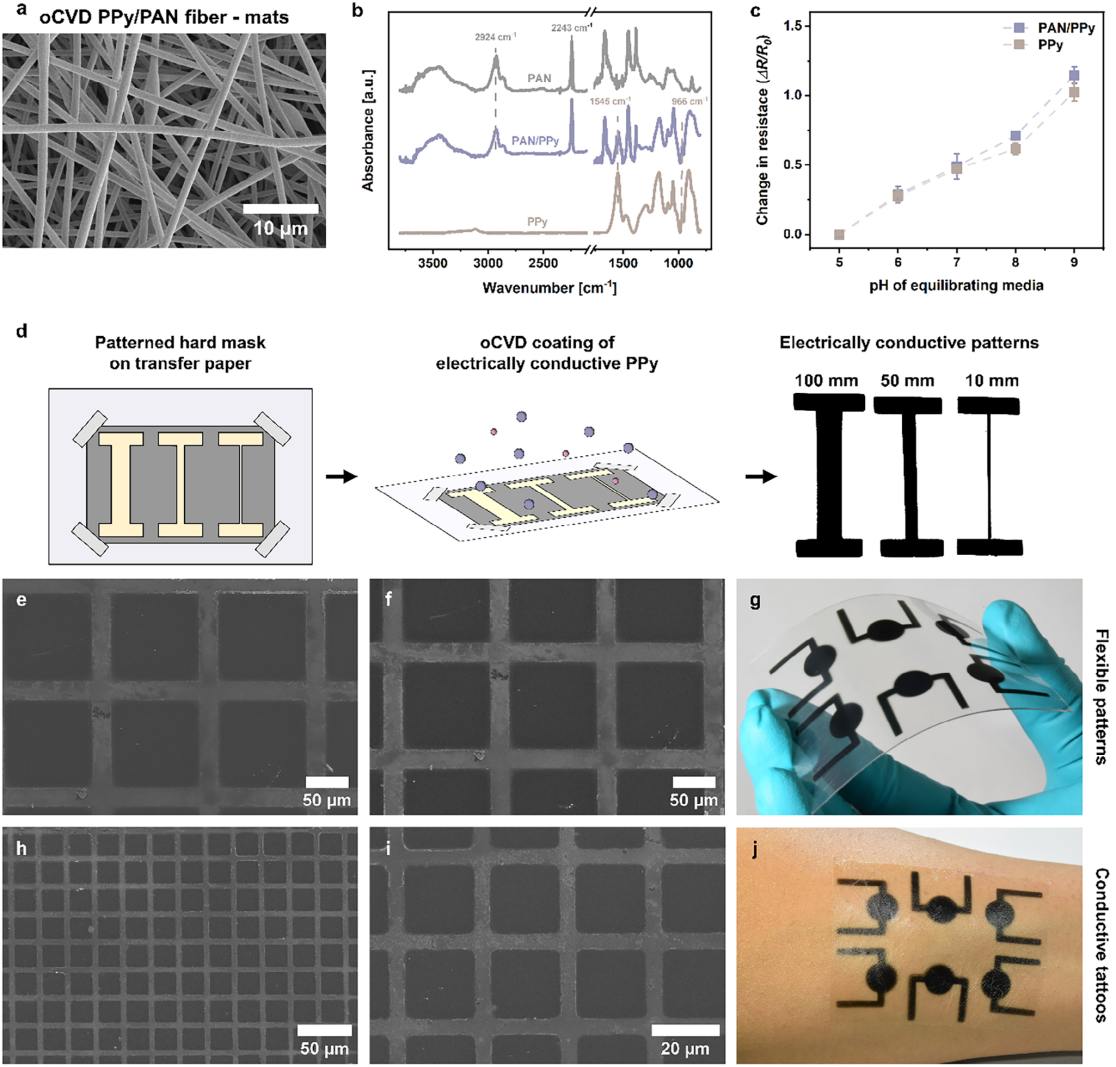
Applicability of oCVD PPy in Fabricating Flexible oCVD Polypyrrole‐coated Electrically Conductive PAN Fiber‐mat pH Sensing Patches and Patterning of Microislands. (a) SEM image of the pH‐responsive oCVD PPy‐coated electrospun PAN fiber mat, demonstrating the conformal coating of individual fibers throughout the cross‐section while preserving the porous microstructure of the fiber mat. (b) FTIR spectra of the oCVD PPy‐coated PAN fiber mat, displaying characteristic peaks of both pure PAN and PPy, confirming successful oCVD. (c) pH‐dependent resistance changes in PAN/PPy and PPy coatings. Both PAN/PPy and PPy samples exhibit similar pH sensitivity, demonstrating consistent pH response while imparting flexibility to the sensor. Error bars represent standard deviation (*n* = 4). (d) Schematic of the fabrication process wherein a patterned shadow mask is secured on transfer paper, followed by oCVD of PPy to develop ultrathin (100 nm) electrically conductive patterns of various feature sizes (10 ‐ 100 mm) without any bleeding, and the resulting patterns can be transferred to flexible substrates (g) for various applications. SEM images of conductive microislands of oCVD PPy on a silicon wafer fabricated using mesh grids with low (e, f) and high density (h, i) of electrically conductive domains. j. Temporary tattoos with monolithically integrated oCVD PPy pH sensors for on‐skin applications such as wound healing monitoring.

To this end, flexible free‐standing PAN fiber mats were electrospun and subsequently coated with the pH‐responsive oCVD PPy layer to fabricate textile‐like pH‐responsive patches, as shown in Figure 6a–c. The microstructure of the pristine electrospun PAN fiber mat (deposition parameters described in Section 4.5.1 were optimized by control experiments) is characterized by randomly oriented fibers (average fiber diameter = 0.5 ± 0.1 µm) with a smooth surface and a high degree of porosity due to the large inter‐fiber distances (shown in Figure S11a,c). The morphology of the oCVD PPy‐coated pH‐responsive PAN fiber mat, shown in Figure 6a, demonstrates that the conformal PPy coatings (thickness = 400 nm) were achieved around individual fibers (indicated by the increase in average fiber diameter to 0.9 ± 0.2 µm as shown in Figures S11b,d,e), preserving the porous microstructure of the pristine electrospun fiber mats. The high porosity of the mats facilitates reactant vapor diffusion throughout the entire structure, enabling uniform coating of individual fibers by the conformal PPy coatings by oCVD, as previously reported in the literature [47, 81, 82]. Moreover, recent studies have demonstrated that electrospun nanofiber mats, with their porous structure and ability to mimic the extracellular matrix, not only enhance breathability but also support cell growth and enable advanced therapeutic and monitoring functions [10].

The presence of characteristic absorption bands uniquely originating from both PAN (*ν_(C‐H)_* and *ν_(C≡N)_*, located at 2924 cm^−1^ and 2243 cm^−1^, respectively) and oCVD PPy (*ν_(C = C/C‐C)_* and *ν_b(C‐N)_*, located at 1545 and 1454 cm^−1^, respectively) in the normalized ATR spectra of the oCVD PPy‐coated PAN fiber mats (PAN/PPy), shown in Figure 6b, further confirms the successful polymerization and deposition of PPy on the electrospun PAN fiber mats via oCVD [68, 83]. The mechanical properties of the electrospun PAN fiber mats before and after oCVD PPy coating were evaluated by uniaxial tensile testing (see Figure S12). Pristine PAN fiber mats exhibit a Young's modulus of 26.28 MPa, a tensile strength of 3.73 MPa, and an elongation at break of 18%. Following oCVD PPy deposition, the PAN/PPy fiber mats show an increased Young's modulus of 33.36 MPa and a slightly higher tensile strength of 3.80 MPa, accompanied by a reduction in elongation at break to 15%. The increase in Young's modulus and tensile strength indicates moderate stiffening and mechanical reinforcement of the electrospun PAN fiber mats, which can be attributed to the presence of the stiff, conformal oCVD PPy coating [47]. This reinforcement is accompanied by a modest reduction in elongation at break, suggesting restricted fiber mobility upon oCVD PPy coating. Importantly, despite this reduction, the PAN/PPy fiber mats retain sufficient mechanical compliance and stretchability, which are critical for flexible and wearable sensing applications. The electrical response curve (Figure 6c) illustrates that the bulk resistance of the pH‐responsive 3D PAN/PPy mat increases with the pH of the equilibrating media, which can be attributed to the deprotonation of the pH‐responsive oCVD PPy chains. This sensitivity is comparable to that of pristine oCVD PPy thin films, which can be partially attributed to the high degree of porosity in the electrically conductive, pH‐responsive oCVD PPy‐coated PAN fiber mats. This porosity allows the equilibrating media to flow throughout the sensing elements, thereby leveraging reversible electrical responses. The stability of the pH‐responsive oCVD PPy‐coated PAN fiber mats was evaluated by monitoring their change in resistance while subjecting them to solutions with pH values cyclically varied between 5, 7, and 8. As shown in Figure S13, the flexible PAN/PPy sensor demonstrates reversible resistance changes in response to these dynamic pH cycles over three consecutive repetitions. These results indicate that oCVD can be effectively combined with advanced fabrication methods such as electrospinning to impart flexibility to the pH sensors without compromising sensitivity and stability.

We further evaluated the array compatibility and patternability of the pH‐responsive PPy coatings via oCVD. Figure 6d illustrates the fabrication process for patterning electrically conductive PPy coatings on arbitrary substrates via oCVD using a shadow mask, where the shadow mask, with an appropriate feature size, was secured onto the substrate using Kapton tape (backside attached to the base to prevent coating), followed by surface coating with the electrically conductive oCVD PPy coatings. Figure 6d demonstrates that the shape fidelity of the conductive oCVD PPy coatings patterned on polycarbonate substrates is maintained across various feature sizes ranging from 100 to 10 mm without any bleeding effects. Furthermore, the oCVD PPy coatings can be patterned into high‐density electrically conductive disconnected micro islands using TEM mesh grids, as depicted in Figure 6e,f (smallest feature size = 100 µm) and Figure 6h,i (smallest feature size = 20 µm). This is not typically attainable by conventional solution polymerization and coating techniques for conjugated polymers such as PPy due to artifacts such as wetting, but is facilitated by the benefits of vapor phase deposition, such as oCVD [84]. Moreover, owing to the substrate‐independent nature of the oCVD process, the electrically conductive PPy coatings were deposited on arbitrary, delicate substrates without significant disadvantages [85]. Figure 6g,j depicts the oCVD PPy coating patterned onto flexible polycarbonate sheets and temporary tattoos with electrically conductive pH sensing elements, respectively, addressing practical challenges in producing a clinical product that is easy to use, disposable, and offers spatiotemporal sensing information [10].

## 3 Conclusions

This study presents the successful fabrication of electrically conductive and biocompatible pH sensors for monitoring dynamic processes, such as wound healing. By employing oxidative chemical vapor deposition (oCVD), ultrathin layers of pH‐responsive polypyrrole (PPy) were deposited on flexible substrates to develop biosensors capable of detecting and responding to changes within the physiologically relevant pH range of 4‐9. Our findings demonstrate that the electrical conductivity of the oCVD PPy pH sensor can be finely tuned by two orders of magnitude, reaching up to 304 S.cm^−1^ at pH 4. GIWAXS studies showed that a higher dopant concentration in oCVD PPy films improves structural ordering and induces an efficient *π–π* stacking, which enhances carrier transport and significantly increases electrical conductivity. Comprehensive spectroscopic analysis (UV–vis–NIR, FTIR, XPS) elucidated the mechanistic changes in the oCVD PPy polymer structure in response to protonation and deprotonation via dopant counterion exchange. This reversible process underlies changes in the electrical resistance of the oCVD PPy thin films. At alkaline pH levels, neutral and polaron band transitions lower conductivity, while at low pH, bipolaron transitions increase it. This reversible doping process allows precise H^+^ ion concentration measurement. The sensor, optimized for thickness and doping concentration, produced linear pH responses from 4 to 9, suitable for wound healing monitoring. The fabricated all‐polymer‐based pH sensor demonstrated high sensitivity and repeatability, with minimal long‐term drift. Real‐life scenario suitability was confirmed by accurately measuring the pH of artificial wound exudates and successive cycles of dynamic changes in acute and chronic wound healing scenarios. Leveraging the substrate‐independent nature of vapor deposition processes such as oCVD, we fabricated flexible pH‐sensitive patches by conformally interfacing the pH response of the oCVD PPy element on porous electrospun PAN nanofiber mats. These mats retained high pH sensitivity while being flexible, addressing several applicative challenges with disposability and compatibility with commercial wound dressings. Furthermore, the doped PPy network can be patterned into electrically conductive microislands using shadow masks. Therefore, the data collectively suggest that oCVD is a promising route for the development of highly integrated designs in conjunction with other readily adaptable fabrication strategies in fabricating flexible pH‐sensing patches for spatiotemporal monitoring of large wound areas, thus advancing the development of advanced wearable bioelectronic devices for wound healing sensing and monitoring. These devices can be readily adopted in conjunction with drug delivery systems to create multiplex theranostic patches, enhancing wound healing monitoring and treatment.

## 4 Materials and Methods

### 4.1 Materials

Pyrrole (Py, C_4_H_5_N, > 99%), antimony pentachloride (SbCl_5_, > 98%), polyacrylonitrile (PAN, *M_w_* = 150,000 g·mol^−1^), dimethylformamide (DMF, HCON(CH_3_)_2_), and sodium hydroxide (NaOH) were procured from Sigma‐Aldrich and utilized as received without further purification. Technical pH buffer solutions (pH 4.01, pH 7, and pH 10) were obtained from Mettler Toledo. IR‐transparent silicon wafers (single‐side polished, prime CZ) were supplied by Sil'tronix, France. Polycarbonate sheets, both transparent and opaque, were sourced from Mayku, London. The temporary transfer tattoo paper used in this study was commercially available and acquired from The Magic Touch Ltd., UK. TEM copper grids (G200 ‐ 200 mesh, G1000HS ‐ 1000 mesh) were purchased from Structure Probe, Inc., USA.

### 4.2 Fabricating oCVD PPy Electrodes via oCVD

The pH‐responsive electrically conductive PPy coating was synthesized via oxidative chemical vapor deposition (oCVD) using vaporized pyrrole (Py) as the monomer and antimony pentachloride (SbCl_5_) as the oxidant in a custom‐built chamber, as detailed in our previous works. To prevent condensation, the reactant gas lines were maintained at 110 °C, while the main reaction chamber was heated to 40 °C. Both reactants were vaporized in glass jars to ensure sufficient vapor pressure for consistent flow during the reaction. The monomer and oxidant flows were precisely controlled at 2.5 sccm and 0.25, 0.50, and 0.75 sccm, respectively, using precision valves, to maintain a predetermined oxidant‐to‐monomer flow ratio (reactant ratio, RR) varying between 0.1 and 0.3. These reactants were introduced into the main reaction chamber via two separate perpendicular inlet ports. A constant nitrogen flow of 15 sccm was used through the oxidant delivery line to act as a diluent and facilitate oxidant flow. The reaction was conducted at a constant pressure of 300 mTorr. The depositions were performed on various substrates, including silicon wafers, Knittel glass, quartz, gold‐patterned glass substrates, polycarbonate sheets, and temporary tattoo paper, leveraging the substrate independence of the oCVD process. The 2D substrates were secured to the base of the main reaction chamber with Kapton tape to facilitate surface coating, but to prevent deposition on the back side of the substrates, and were maintained at 40 °C throughout the process. For patterned oCVD PPy coatings, appropriate hard masks were affixed to the substrates using Kapton tape, and micropatterned PPy coatings were achieved by securing copper TEM grids onto silicon wafers. To determine the deposition rate and target film thickness, a calibration curve was established by depositing oCVD PPy (RR = 0.2) on pristine silicon wafers for varying deposition times. The resulting film thicknesses were measured using stylus profilometry (Bruker DektakXT), and thickness was plotted as a function of deposition time (Figure S7). Based on the calibration curve, subsequent oCVD depositions were performed for appropriate durations to achieve PPy film thicknesses of approximately 100 nm. Under these conditions, a typical deposition cycle of 30 min followed by 30 min of nitrogen degassing resulted in PPy coatings with a thickness of 102 ± 8 nm.

### 4.3 Characterization

Borosilicate glass substrates were patterned with Cr (5 nm as an adhesion layer) and Au (40 nm) via thermal evaporation using a shadow mask. The resulting parallel line‐shape electrodes had a width (*w*) of 4.5mm and a channel length (*l*) of 1mm. The oCVD PPy‐coated samples were then immersed in solutions with pH values ranging from 4 to 12, under solvent‐abundant conditions, for 5 min. Solutions with pH values from 4 to 10 were prepared by blending appropriate amounts of standard technical pH buffer solutions (pH 4.01, pH 7, and pH 10), while solutions of pH 11 and 12 were generated by adding the requisite amount of 0.5 M NaOH stock solution to the standard pH 10 buffer solution. Additionally, oCVD PPy‐coated samples were equilibrated in an artificial wound exudate adjusted to pH 6.3, 7.2, and 8.5. The artificial wound exudates were used as obtained by mixing Dulbecco's modified Eagle medium (DMEM) with 10% fetal bovine serum (FBS) and 1% Pen Strep, resulting in a pH of 7.2. The pH of the solution was adjusted to 6.3 and 8.5 using appropriate amounts of HCl and NaOH. Following equilibration, the samples were dried using Kimtech wipes, and their electrical resistance was measured in an inert atmosphere using a Keithley 4200 SCS parameter analyzer using a 4‐point probe configuration. The electrical conductivity was then calculated from the measured electrical resistance using Equation (1). Reported electrical conductivity values represent averages of four repetitions.

(1) $$\sigma =\frac{l}{R*w*t}\;$$

where *l* = length of electrode = 1mm, *w* = width of the electrode = 4.5 mm, *t* = thickness of the oCVD PPy coating, *R* = measured in‐plane resistance of the material, and σ = electrical conductivity of the material.

UV—vis–NIR spectra of oCVD PPy coatings on glass substrates were acquired via absorption measurements using a Shimadzu spectrometer (UV‐3600 UV—vis–NIR). Samples were equilibrated in different pH solutions ranging from 4 to 12 as previously described. Subsequently, spectra of the dried samples were collected in the range of 350 ‐ 2000 nm, with pristine glass serving as the background. Peak deconvolution of the absorbance spectra from UV‐vis‐NIR measurements was conducted by fitting Gaussian functions using Origin 2018 software.

To determine the optical bandgap of oCVD PPy coatings equilibrated in pH 4–12, UV–vis spectroscopy was performed on oCVD PPy‐coated quartz substrates utilizing an Agilent Cary 60 UV‐vis spectrophotometer. Absorbance data were collected from 190 ‐ 1100 nm, with pristine quartz as the background.

X‐ray photoelectron spectroscopy (XPS) analysis was conducted on oCVD PPy coatings on glass substrates equilibrated in solutions of pH 5, 8, and 11, and subsequently dried. Measurements were performed using an untreated oCVD PPy film as a reference, employing a Surface Science SSX‐100 ESCA instrument with a monochromatic Al Kα X‐ray source (*hν* = 1486.6 eV). The pressure in the analysis chamber was maintained below 5 × 10^−9^ mbar. The electron take‐off angle was set to 37 ° relative to the surface normal, and the analyzed spot had a diameter of 1000 µm. To mitigate charging effects, a gold grid positioned approximately 1 mm above the sample generated secondary electrons to neutralize the positive charge on the surface after photoemission. Survey spectra and detailed spectra of the C1*s*, N1*s*, Cl2*p*, and Sb*3d* core levels were recorded with an energy resolution of 1.3 eV. The sp2 C1*s* photoemission peak of the PPy coatings at a binding energy (BE) of 284.2 eV [38] served as the reference for the binding energies, with reported binding energies having an accuracy of ± 0.1 eV. XPS data analysis was performed using the least‐squares curve‐fitting program, Winspec (LISE laboratory, University of Namur, Belgium), involving subtraction of a Shirley baseline and fitting with a minimum number of peaks, where the peak profile was a convolution of Gaussian and Lorentzian functions. To assess sample uniformity, measurements were taken at two distinct spots on each sample, with the uncertainty in peak intensity determination within 2% for all reported core levels.

The chemical composition of all oCVD PPy films deposited on IR‐transparent silicon wafers (untreated and equilibrated in pH solutions 4–12) was investigated by Fourier‐transform infrared spectroscopy (FTIR) using a Shimadzu IRTracer in absorbance mode. Spectra were collected over a wavenumber range of 500–4000 cm^−1^ with a resolution of 4 cm^−1^ and averaged over 128 scans, utilizing a bare silicon wafer as the background. Standard baseline correction and normalization procedures were performed on the spectra using Origin 2018 software.

The surface morphology of micropatterned electrically conductive oCVD PPy domains was examined using scanning electron microscopy (SEM) with a Nova NanoSEM 650. The SEM was operated at an acceleration voltage of 15 kV, and images were captured from a working distance of 5 mm. The size of the domains was determined using ImageJ software, and the average was measured over 20 measurements.

The conformality of the oCVD PPy‐based temporary tattoos to human skin was evaluated by affixing the temporary conductive tattoo onto a participant solely for imaging to demonstrate mechanical conformability, and written informed consent was obtained from the individual appearing in the image.

The stability of the oCVD PPy‐based pH‐sensing films was evaluated by cyclic convex bending tests. The samples were mounted on custom‐designed bending fixtures imposing bending strains of 0.5% and 1.0%. During bending, the oCVD PPy layer was positioned on the outer side of the bend and thus subjected to tensile strain. The sensors were subjected to 300 bending cycles at each strain level under ambient conditions. The electrical resistance of the oCVD PPy sensing layer was recorded after cycling using a digital multimeter, and the relative change in resistance was calculated to assess stability.

Grazing Incidence Wide‐Angle X‐Rays Scattering (GIWAXS) measurements were carried out at the multipurpose instrument for nanostructural analysis (MINA) of the University of Groningen, equipped with a Cu rotating anode emitting X‐ray wavelength at 1.5413 Å (corresponding to 8 keV). 2D patterns were collected using a Bruker Vantec 500 2D detector (1024 × 1024 pixel array with a pixel size of 136 × 136 µm) located 96 mm away from the sample. The oCVD polypyrrole thin films supported on silicon wafers were placed in reflection geometry at 0.2° incident angle with respect to the direct beam. The direct beam center position on the detector and the sample‐to‐detector distance were calibrated using the diffraction rings from standard silver behenate powder. All the necessary corrections for the GIWAXS geometry were applied to the raw patterns using the GIXSGUI MATLAB toolbox [86]. The reshaped GIWAXS patterns, taking into account the inaccessible part in reciprocal space (wedge‐shaped corrected patterns), are presented as a function of the quasi‐vertical and parallel scattering vectors *q_z_* and *q_r_*.

### 4.4 pH Sensing

A pH sensor strip was prepared by coating a polycarbonate strip (4×1 cm^2^) with a circular pattern of the pH‐responsive oCVD PPy layer (0.5 cm diameter, 100 nm thickness) using a shadow mask. The resistance across the oCVD PPy layer was measured by attaching copper wires as electrodes on either side of the sensing layer, utilizing a modular digital multimeter (Keysight BenchVue U2741A). The pH sensing strip was then sandwiched between two plates of a custom‐built cell (shown in Figure S9), where the oCVD PPy sensing layer was exposed to various pH buffer solutions (100 µL) through an aperture on the top. The electrical resistance of the pH sensor was measured after an equilibration time of 5 min, and the average values were reported.

### 4.5 pH‐Responsive oCVD PPy/PAN Patches

#### 4.5.1 Fabrication

pH‐responsive patches were engineered through the oCVD deposition of PPy on free‐standing electrospun PAN fiber mats. Initially, PAN pellets (10 w.v^−1^%) were dissolved in DMF and stirred overnight at 60°C to yield a colorless, homogeneous precursor solution for electrospinning. Electrospinning of the fiber mats was conducted using an Inovenso NanoSpinner NE300 electrospinning machine under ambient conditions. The precursor solution was loaded into a plastic syringe equipped with an 18 G needle connected to a syringe pump, operating at a volume flow rate of 0.5 mL.h^−1^. A flat collector plate, wrapped in aluminum foil, served to collect the fiber mats. During electrospinning, the tip‐collector distance was maintained at 10 cm, and a voltage of 11 kV was applied, optimized through calibration experiments. The electrospinning process was continued until free‐standing PAN fiber mats with a thickness of approximately 100 µm could be detached from the aluminum foil for subsequent processing. Following electrospinning, the free‐standing PAN fiber mats served as substrates for oCVD of PPy to deposit the pH‐responsive layer, as outlined in Section 4.2. Building upon our previous work, a custom‐built stage was employed to ensure uniform and conformal coating of individual fibers throughout the PAN fiber mat substrates [47]. These mats were oriented perpendicular to the flows of reactant gases‐ using a custom‐built sample holder, aligned with the monomer flow to minimize direct exposure to the oxidant and facilitate simultaneous coating from both sides.

#### 4.5.2 Characterization

The morphology of the pristine (for electrospinning calibration experiments) and the PPy‐coated PAN fiber mats was observed by SEM (Nova NanoSEM 650) with a working distance of 5 mm and an acceleration voltage of 10 kV. To avoid charging effects, the pristine PAN fiber mats were coated with 5 nm of gold before making observations. The mean fiber diameter of the uncoated and oCVD PPy‐coated PAN fiber mats was calculated by performing a fiber diameter analysis (averaging over 100 fibers) using ImageJ software.

The chemical composition of all the uncoated and the oCVD PPy‐coated PAN fiber mats was investigated by ATR (Shimadzu IRTracer) in absorbance mode. The spectra were collected over a wavenumber range of 800–3600 cm^−1^ with a resolution of 4 cm^−1^ and averaged over 128 scans. The FTIR spectra of pristine oCVD PPy have been used for comparison.

The mechanical performance of the electrospun PAN fiber mats and oCVD PPy‐coated PAN fiber mats was evaluated by uniaxial tensile testing to failure using an Instron 68SC‐1 tensile tester at a constant crosshead speed of 20 mm min^−1^. Young's modulus, tensile strength, and elongation at break were determined from the resulting stress‐strain curves and averaged over three independent samples.

The electrical response of the PAN/PPy pH sensor was evaluated by measuring the bulk resistance of the fiber mat using a modular digital multimeter (Keysight BenchVue U2741A) and attaching copper electrodes to the ends of the fiber mat. The relative change in the electrical resistance was measured after equilibrating the PAN/PPy fiber mat in solutions of varying pH between 5‐9, as well as repeated cycles between pH 5–7 and 5–8. All samples were immersed for 5 min to ensure reproducibility.

## Author Contributions

The manuscript was written through the contributions of all authors. All authors have approved the final version of the manuscript.

## Funding

M.K. and J.S. gratefully acknowledge the European Research Council (ERC) for the financial support under the European Union's Horizon 2020 research and innovation program under the Consolidator grant agreement no. 864982. A.M., R.K.B., M.K., and A.G.P.K. acknowledge the PhD Scholarship Program issued by the Dutch Ministry of Education, Culture, and Science (OCW) within the framework of the national PhD Scholarship Programme Experiment.

## Conflicts of Interest

The authors declare no conflicts of interest.

## Supporting information




**Supporting File**: adhm70891‐sup‐0001‐SuppMat.docx.

## Data Availability

Data for this article are available at DataverseNL at DOI/URL https://doi.org/10.34894/KSWCPE.

## References

[1] C. Wang , E. Shirzaei Sani , and W. Gao , “Wearable Bioelectronics for Chronic Wound Management,” Advanced Functional Materials 32, no. 17 (2022): 2111022, 10.1002/adfm.202111022. 36186921 PMC9518812

[2] Y. Luo , M. R. Abidian , J.‐H. Ahn , et al., “Technology Roadmap for Flexible Sensors,” ACS Nano 17, no. 6 (2023): 5211–5295, 10.1021/acsnano.2c12606. 36892156 PMC11223676

[3] A. McLister , J. McHugh , J. Cundell , and J. Davis , “New Developments in Smart Bandage Technologies for Wound Diagnostics,” Advanced Materials 28, no. 27 (2016): 5732–5737, 10.1002/adma.201504829. 26821765

[4] H. Kim , J. H. Kim , M. Jeong , et al., “Bioelectronic Sutures with Electrochemical pH‐Sensing for Long‐Term Monitoring of the Wound Healing Progress,” Advanced Functional Materials 34, no. 40 (2024): 2402501, 10.1002/adfm.202402501.

[5] Q. Zhong , R. Zhang , Y. Chen , et al., “A Mechanical Contraction‐Driven Hydrogel Dressing for pH Visualization and Tailored Acute/Chronic Wound Healing,” Advanced Functional Materials (2025): 12807, 10.1002/adfm.202512807.

[6] X. Gong , J. Yang , Y. Zheng , et al., “Polymer Hydrogel‐Based Multifunctional Theranostics for Managing Diabetic Wounds,” Advanced Functional Materials 34, no. 26 (2024): 2315564, 10.1002/adfm.202315564.

[7] Y. Ling , T. An , L. W. Yap , et al., “Disruptive, Soft, Wearable Sensors,” Advanced Materials 32, no. 18 (2020): 1904664, 10.1002/adma.201904664. 31721340

[8] G. Xu , Y. Lu , C. Cheng , et al., “Battery‐Free and Wireless Smart Wound Dressing for Wound Infection Monitoring and Electrically Controlled On‐Demand Drug Delivery,” Advanced Functional Materials 31, no. 26 (2021): 2100852, 10.1002/adfm.202100852.

[9] G. Power , Z. Moore , and T. O'Connor , “Measurement of pH, Exudate Composition and Temperature in Wound Healing: A Systematic Review,” Journal of Wound Care 26, no. 7 (2017): 381–397, 10.12968/jowc.2017.26.7.381. 28704150

[10] S. O'Callaghan , P. Galvin , C. O'Mahony , Z. E. H. Moore , and R. Derwin , “‘Smart’ Wound Dressings for Advanced Wound Care: A review,” Journal of wound care 29, no. 7 (2020): 394–406, 10.12968/jowc.2020.29.7.394. 32654609

[11] P. Kassal , J. Kim , R. Kumar , et al., “Smart Bandage with Wireless Connectivity for Uric Acid Biosensing as an Indicator of Wound Status,” Electrochemistry Communications 56 (2015): 6–10, 10.1016/j.elecom.2015.03.018.

[12] L. Meng , S. Liu , B. A. Borsa , M. Eriksson , and W. C. Mak , “A Conducting Polymer‐based Array with Multiplex Sensing and Drug Delivery Capabilities for Smart Bandages,” Communications Materials 5, no. 1 (2024): 28, 10.1038/s43246-024-00469-5.

[13] M. Ochoa , R. Rahimi , J. Zhou , et al., “Integrated Sensing and Delivery of Oxygen for Next‐Generation Smart Wound Dressings,” Microsystems & Nanoengineering 6, no. 1 (2020): 46, 10.1038/s41378-020-0141-7. 34567658 PMC8433317

[14] K. Wang , Q. Ding , M. Qi , et al., “Integrated Bilayer Microneedle Dressing and Triboelectric Nanogenerator for Intelligent Management of Infected Wounds,” Advanced Functional Materials 34, no. 28 (2024): 2316820, 10.1002/adfm.202316820.

[15] W. Huang , Y. Xu , Y. Yang , et al., “Wearable Sensor for Continuous Monitoring Multiple Biofluids: Improved Performances by Conductive Metal‐Organic Framework with Dual‐Redox Sites on Flexible Graphene Fiber Microelectrode,” Advanced Functional Materials 35, no. 31 (2025): 2424018, 10.1002/adfm.202424018.

[16] S. RoyChoudhury , Y. Umasankar , J. Jaller , et al., “Continuous Monitoring of Wound Healing Using a Wearable Enzymatic Uric Acid Biosensor,” Journal of The Electrochemical Society 165, no. 8 (2018): B3168, 10.1149/2.0231808jes.

[17] H. Schade and A. Marchionini , “Zur Physikalischen Chemie Der Hautoberfläche,” Archiv für Dermatologie und Syphilis 154, no. 3 (1928): 690–716, 10.1007/BF01828330.

[18] K. Fukuda , Y. Ito , Y. Furuichi , et al., “Three Stepwise pH Progressions in Stratum Corneum for Homeostatic Maintenance of the Skin,” Nature Communications 15, no. 1 (2024): 4062, 10.1038/s41467-024-48226-z. PMC11096370 38750035

[19] J. W. Fluhr and P. M. Elias , “Stratum corneum pH: Formation and Function of the ‘Acid Mantle’,” Exogenous Dermatology 1, no. 4 (2002): 163–175, 10.1159/000066140.

[20] C. R. Kruse , M. Singh , S. Targosinski , et al., “The Effect of pH on Cell Viability, Cell Migration, Cell Proliferation, Wound Closure, and Wound Reepithelialization: In Vitro and in Vivo Study,” Wound Repair and Regeneration 25, no. 2 (2017): 260–269, 10.1111/wrr.12526. 28370923

[21] L. P. Tricou , M. L. Al‐Hawat , K. Cherifi , G. Manrique , B. R. Freedman , and S. Matoori , “Wound pH‐Modulating Strategies for Diabetic Wound Healing,” Advances in Wound Care (New Rochelle) 13 (2024): 446–462, 10.1089/wound.2023.0129. PMC11535470 38149883

[22] Z. Xu , J. Fan , W. Tian , et al., “Cellulose‐Based pH‐Responsive Janus Dressing with Unidirectional Moisture Drainage for Exudate Management and Diabetic Wounds Healing,” Advanced Functional Materials 34, no. 3 (2024): 2307449, 10.1002/adfm.202307449.

[23] S. L. Percival , S. McCarty , J. A. Hunt , and E. J. Woods , “The Effects of pH on Wound Healing, Biofilms, and Antimicrobial Efficacy,” Wound Repair and Regeneration 22, no. 2 (2014): 174–186, 10.1111/wrr.12125. 24611980

[24] P. Sim , X. L. Strudwick , Y. Song , A. J. Cowin , and S. Garg , “Influence of Acidic pH on Wound Healing in Vivo: A Novel Perspective for Wound Treatment,” International Journal of Molecular Sciences 23, no. 21 (2022): 13655, 10.3390/ijms232113655. 36362441 PMC9658872

[25] Y. Liu , X. Luo , L. Chen , Z. Qiao , C. Si , and X. Liu , “Dual‐Layer Modular Microneedle‐Based Biosensor for Advanced Precision Management of Infected Chronic Wounds,” Advanced Functional Materials (2025): 17521.

[26] S. Cho , J.‐H. Ha , J. Ahn , et al., “Wireless, Battery‐Free, Optoelectronic Diagnostic Sensor Integrated Colorimetric Dressing for Advanced Wound Care,” Advanced Functional Materials 34, no. 29 (2024): 2316196, 10.1002/adfm.202316196.

[27] A. Tamayol , M. Akbari , Y. Zilberman , et al., “Flexible pH‐Sensing Hydrogel Fibers for Epidermal Applications,” Advanced Healthcare Materials 5, no. 6 (2016): 711–719, 10.1002/adhm.201500553. 26799457 PMC4805432

[28] K. Unger , F. Greco , and A. M. Coclite , “Temporary Tattoo pH Sensor with pH‐Responsive Hydrogel via Initiated Chemical Vapor Deposition,” Advanced Materials Technologies 7, no. 5 (2022): 2100717, 10.1002/admt.202100717.

[29] Y. Zhu , J. Zhang , J. Song , et al., “A Multifunctional Pro‐Healing Zwitterionic Hydrogel for Simultaneous Optical Monitoring of pH and Glucose in Diabetic Wound Treatment,” Advanced Functional Materials 30, no. 6 (2020): 1905493, 10.1002/adfm.201905493.

[30] Y. ElSaboni , J. A. Hunt , J. Stanley , C. Moffatt , and Y. Wei , “Development of a Textile Based Protein Sensor for Monitoring the Healing Progress of a Wound,” Scientific Reports 12, no. 1 (2022): 7972, 10.1038/s41598-022-11982-3. 35562402 PMC9106706

[31] K. Lim , H. Seo , W. G. Chung , et al., “Material and Structural Considerations for High‐performance Electrodes for Wearable Skin Devices,” Communications Materials 5, no. 1 (2024): 49, 10.1038/s43246-024-00490-8.

[32] P. Gutruf , “Towards a Digitally Connected Body for Holistic and Continuous Health Insight,” Communications Materials 5, no. 1 (2024): 2, 10.1038/s43246-023-00443-7.

[33] P. Nayak , N. Kurra , C. Xia , and H. N. Alshareef , “Highly Efficient Laser Scribed Graphene Electrodes for On‐Chip Electrochemical Sensing Applications,” Advanced Electronic Materials 2, no. 10 (2016): 1600185, 10.1002/aelm.201600185.

[34] X. Chen , Y. J. Park , M. Kang , et al., “CVD‐grown Monolayer MoS2 in Bioabsorbable Electronics and Biosensors,” Nature communications 9, no. 1 (2018): 1690. 10.1038/s41467-018-03956-9 PMC5924366 29703901

[35] C. Gu , L. Zhang , T. Hou , Q. Wang , F. Li , and P. Gai , “Laser‐Induced Nanozyme Biofuel Cell‐Based Self‐Powered Patch for Accelerating Diabetic Wound Healing With Real‐Time Monitoring,” Advanced Functional Materials 35, no. 32 (2025): 2423106, 10.1002/adfm.202423106.

[36] M. Asaduzzaman , O. Faruk , A. A. Samad , et al., “A MOFs‐Derived Hydroxyl‐Functionalized Hybrid Nanoporous Carbon Incorporated Laser‐Scribed Graphene‐Based Multimodal Skin Patch for Perspiration Analysis and Electrocardiogram Monitoring,” Advanced Functional Materials 34, no. 40 (2024): 2405651, 10.1002/adfm.202405651.

[37] E. Shirzaei Sani , C. Xu , C. Wang , et al., “A Stretchable Wireless Wearable Bioelectronic System for Multiplexed Monitoring and Combination Treatment of Infected Chronic Wounds,” Science Advances 9, no. 12: adf7388, 10.1126/sciadv.adf7388. PMC10038347 36961905

[38] T. Wang , Y. Zhang , Q. Liu , et al., “A Self‐Healable, Highly Stretchable, and Solution Processable Conductive Polymer Composite for Ultrasensitive Strain and Pressure Sensing,” Advanced Functional Materials 28, no. 7 (2018): 1705551, 10.1002/adfm.201705551.

[39] W. Wang , P. Guo , X. Liu , et al., “Fully Polymeric Conductive Hydrogels with Low Hysteresis and High Toughness as Multi‐Responsive and Self‐Powered Wearable Sensors,” Advanced Functional Materials 34, no. 32 (2024): 2316346, 10.1002/adfm.202316346.

[40] Y. Qin , X. Qu , B. Huang , et al., “In Vivo Synthesis of Metabolically Degradable π‑Conjugated Conductive Polymers Enabling Seamless Neural Interface Integration and Tissue Repair,” Advanced Functional Materials 35, no. 37 (2025): 2501813, 10.1002/adfm.202501813.

[41] Z. Fan , T. Xue , Y. Mu , et al., “Synergistic Enhancement of Zinc‐Ion Hybrid Capacitors via Redox‐Active Doping and Sulfonated MXene‐Modified Polypyrrole Cathodes,” Advanced Functional Materials 35 (2025): 10767, 10.1002/adfm.202510767.

[42] Y. Song , T.‐Y. Liu , X.‐X. Xu , D.‐Y. Feng , Y. Li , and X.‐X. Liu , “Pushing the Cycling Stability Limit of Polypyrrole for Supercapacitors,” Advanced Functional Materials 25, no. 29 (2015): 4626–4632, 10.1002/adfm.201501709.

[43] X. Tan , C. Hu , Z. Zhu , H. Liu , and J. Qu , “Electrically Pore‐Size‐Tunable Polypyrrole Membrane for Antifouling and Selective Separation,” Advanced Functional Materials 29, no. 35 (2019): 1903081, 10.1002/adfm.201903081.

[44] J. H. Kaufman , N. Colaneri , J. C. Scott , and G. B. Street , “Evolution of Polaron States into Bipolarons in Polypyrrole,” Physical Review Letters 53, no. 10 (1984): 1005–1008, 10.1103/PhysRevLett.53.1005.

[45] Z. Zhou , M. Liu , H. You , et al., “Multiple‐Stimuli‐Responsive Biomimetic Polypyrrole Delivery System for Synergistic Regulation and Restoration of Epileptic Foci,” Advanced Functional Materials 35 (2025): 10305, 10.1002/adfm.202510305.

[46] A. Dianatdar , A. Mukherjee , and R. K. Bose , “Oxidative Chemical Vapor Deposition of Polypyrrole onto Carbon Fabric for Flexible Supercapacitive Electrode Material,” Synthetic Metals 298 (2023): 117444, 10.1016/j.synthmet.2023.117444.

[47] A. Mukherjee , A. Dianatdar , M. Z. Gładysz , et al., “Electrically Conductive and Highly Stretchable Piezoresistive Polymer Nanocomposites via Oxidative Chemical Vapor Deposition,” ACS Applied Materials & Interfaces 15, no. 26 (2023): 31899–31916, 10.1021/acsami.3c06015. 37345686 PMC10326852

[48] A. Dianatdar , M. Miola , O. De Luca , P. Rudolf , F. Picchioni , and R. K. Bose , “All‐dry, One‐step Synthesis, Doping and Film Formation of Conductive Polypyrrole,” Journal of Materials Chemistry C 10, no. 2 (2022): 557–570, 10.1039/D1TC05082F.

[49] H. Ullah , A.‐U.‐H. A. Shah , S. Bilal , and K. Ayub , “Doping and Dedoping Processes of Polypyrrole: DFT Study with Hybrid Functionals,” The Journal of Physical Chemistry C 118, no. 31 (2014): 17819–17830, 10.1021/jp505626d.

[50] Q. Pei and R. Qian , “Protonation and Deprotonation of Polypyrrole Chain in Aqueous Solutions,” Synthetic Metals 45, no. 1 (1991): 35–48, 10.1016/0379-6779(91)91845-2.

[51] I. L. Lehr , O. V. Quinzani , and S. B. Saidman , “Comparative Study of Polypyrrole Films Electrosynthesized in Alkaline and Acid Solutions,” Materials Chemistry and Physics 117, no. 1 (2009): 250–256, 10.1016/j.matchemphys.

[52] A. Dianatdar and R. Bose , “Oxidative Chemical Vapor Deposition for Synthesis and Processing of Conjugated Polymers: A Critical Review,” Journal of Materials Chemistry C 11 (2023): 11776–11802, 10.1039/D3TC01614E.

[53] A. Shukla , D. Das , and K. Sen , “Electrically‐assisted chemical vapor polymerization: A novel method for in situ polymerization of pyrrole,” Journal of Applied Polymer Science 140, no. 6 (2023): 53443, 10.1002/app.53443.

[54] J. M. Ribó , M. C. Anglada , J. M. Tura , and N. Ferrer‐Anglada , “Comparison of Polypyrrole, Poly(3,4‐dimethylpyrrole) and Poly(3‐methoxy‐4‐methylpyrrole),” Synthetic Metals 72, no. 2 (1995): 173–176, 10.1016/0379-6779(94)02325-S.

[55] Y. Yamashita , J. Tsurumi , M. Ohno , et al., “Efficient Molecular Doping of Polymeric Semiconductors Driven by Anion Exchange,” Nature 572, no. 7771 (2019): 634–638, 10.1038/s41586-019-1504-9. 31462795

[56] J. Park , Y. Lee , M. Kim , et al., “Closely Packed Polypyrroles via Ionic Cross‐Linking: Correlation of Molecular Structure–Morphology–Thermoelectric Properties,” ACS Applied Materials & Interfaces 12, no. 1 (2020): 1110–1119, 10.1021/acsami.9b17009. 31825593

[57] R. G. Davidson , L. C. Hammond , T. G. Turner , and A. R. Wilson , “An Electron and X‐ray Diffraction Study of Conducting Polypyrrole/Dodecyl Sulfate,” Synthetic Metals 81, no. 1 (1996): 1–4, 10.1016/0379-6779(96)80221-9.

[58] J. Dong and G. Portale , “Role of the Processing Solvent on the Electrical Conductivity of PEDOT:PSS,” Advanced Materials Interfaces 7, no. 18 (2020): 2000641, 10.1002/admi.202000641.

[59] J. Edberg , D. Iandolo , R. Brooke , et al., “Patterning and Conductivity Modulation of Conductive Polymers by UV Light Exposure,” Advanced Functional Materials 26, no. 38 (2016): 6950–6960, 10.1002/adfm.201601794.

[60] O. Bubnova , Z. U. Khan , A. Malti , et al., “Optimization of the thermoelectric figure of merit in the conducting polymer poly(3,4‐ethylenedioxythiophene),” Nature Materials 10, no. 6 (2011): 429–433, 10.1038/nmat3012. 21532583

[61] K. Yakushi , L. J. Lauchlan , T. C. Clarke , and G. B. Street , “Optical Study of Polypyrrole Perchlorate,” The Journal of Chemical Physics 79, no. 10 (1983): 4774–4778, 10.1063/1.445621.

[62] D. A. Chesher , P. A. Christensen , and A. Hamnett , “Anion Movement and Carrier Type in Polypyrrole/Dodecyl Sulfate,” Journal of the Chemical Society, Faraday Transactions 89, no. 2 (1993): 303–309, 10.1039/FT9938900303.

[63] P. A. Christensen and A. Hamnett , “In Situ Spectroscopic Investigations of the Growth, Electrochemical Cycling and Overoxidation of Polypyrrole in Aqueous Solution,” Electrochimica Acta 36, no. 8 (1991): 1263–1286, 10.1016/0013-4686(91)80005-S.

[64] F. Devreux , F. Genoud , M. Nechtschein , and B. Villeret , “ESR Investigation of Polarons and Bipolarons in Conducting Polymers:: the Case of Polypyrrole,” Synthetic Metals 18, no. 1 (1987): 89–94, 10.1016/0379-6779(87)90859-9.

[65] F. Genoud , M. Guglielmi , M. Nechtschein , E. Genies , and M. Salmon , “ESR Study of Electrochemical Doping in the Conducting Polymer Polypyrrole,” Physical Review Letters 55, no. 1 (1985): 118–121, 10.1103/PhysRevLett.55.118. 10031695

[66] J. L. Brédas , B. Thémans , J. G. Fripiat , J. M. André , and R. R. Chance , “Highly Conducting Polyparaphenylene, Polypyrrole, and Polythiophene Chains: An Ab Initio Study of the Geometry and Electronic‐structure Modifications Upon Doping,” Physical Review B 29, no. 12 (1984): 6761–6773, 10.1103/PhysRevB.29.6761.

[67] P. Makuła , M. Pacia , and W. Macyk , “How To Correctly Determine the Band Gap Energy of Modified Semiconductor Photocatalysts Based on UV‐Vis Spectra,” The Journal of Physical Chemistry Letters 9, no. 23 (2018): 6814–6817, 10.1021/acs.jpclett.8b02892. 30990726

[68] B. Tian and G. Zerbi , “Lattice Dynamics and Vibrational Spectra of Pristine and Doped Polypyrrole: Effective Conjugation Coordinate,” The Journal of Chemical Physics 92, no. 6 (1990): 3892–3898, 10.1063/1.457795.

[69] B. Tian and G. Zerbi , “Lattice Dynamics and Vibrational Spectra of Polypyrrole,” The Journal of Chemical Physics 92, no. 6 (1990): 3886–3891, 10.1063/1.457794.

[70] G. Zerbi , M. Gussoni , and C. Castiglioni , “Vibrational Spectroscopy of Polyconjugated Aromatic Materials with Electrical and Non Linear Optical Properties,” in Conjugated Polymers: The Novel Science and Technology of Highly Conducting and Nonlinear Optically Active Materials, ed. J. L. Brédas and R. Silbey (Springer, 1991), 435–507, 10.1007/978-94-011-3476-7_10.

[71] J. Tabačiarová , M. Mičušík , P. Fedorko , and M. Omastová , “Study of Polypyrrole Aging by XPS, FTIR and Conductivity Measurements,” Polymer Degradation and Stability 120 (2015): 392–401, 10.1016/j.polymdegradstab.2015.07.021.

[72] R. G. Davidson and T. G. Turner , “An IR Spectroscopic Study of the Electrochemical Reduction of Polypyrrole Doped with Dodecylsulfate Anion,” Synthetic Metals 72, no. 2 (1995): 121–128, 10.1016/0379-6779(94)02332-S.

[73] J. Stejskal , M. Trchová , P. Bober , et al., “Polypyrrole Salts and Bases: Superior Conductivity of Nanotubes and Their Stability towards the Loss of Conductivity by Deprotonation,” RSC Advances 6, no. 91 (2016): 88382–88391, 10.1039/C6RA19461C.

[74] C. Malitesta , I. Losito , L. Sabbatini , and P. G. Zambonin , “New Findings on Polypyrrole Chemical Structure by XPS Coupled to Chemical Derivatization Labelling,” Journal of Electron Spectroscopy and Related Phenomena 76 (1995): 629–634, 10.1016/0368-2048(95)02438-7.

[75] B. Saoudi , N. Jammul , M. M. Chehimi , A.‐S. Jaubert , C. Arkam , and M. Delamar , “XPS Study of the Adsorption Mechanisms of DNA onto Polypyrrole Particles,” Spectroscopy 18 (2004): 943729, 10.1155/2004/943729.

[76] Y. Laffitte and B. L. Gray , “Potentiometric pH Sensor Based on Flexible Screen‐Printable Polyaniline Composite for Textile‐Based Microfluidic Applications,” Micromachines 13, no. 9 (2022): 1376, 10.3390/mi13091376. 36143999 PMC9503819

[77] Y. Li , Y. Mao , C. Xiao , X. Xu , and X. Li , “Flexible pH Sensor Based on a Conductive PANI Membrane for pH Monitoring,” RSC Advances 10, no. 1 (2020): 21–28, 10.1039/c9ra09188b. PMC9047031 35492551

[78] M. S. Hossain , N. Padmanathan , M. M. R. Badal , K. M. Razeeb , and M. Jamal , “Highly Sensitive Potentiometric pH Sensor Based on Polyaniline Modified Carbon Fiber Cloth for Food and Pharmaceutical Applications,” ACS Omega 9, no. 38 (2024): 40122–40133, 10.1021/acsomega.4c06090. 39346860 PMC11425811

[79] A. E. Ávila Ramírez , D. P. van der Laan , M. B. Shah , L. Wang , E. Zeglio , and A. Savva , “PEDOT:PSS—A Key Material for Bioelectronics,” Advanced Science (2025): 13480, 10.1002/advs.202513480. PMC12931241 41431935

[80] C. A. Megchum‐Ruedas , P. M. Velasco‐Bolom , R. Grajales‐Coutiño , J. L. Camas‐Anzueto , M. Pérez‐Patricio , and C. A. Hernández‐Gutiérrez , “A Film Composed of PEDOT:PSS/PVA as a Sensitive Medium for pH Sensor in Optical Fiber,” Measurement 233 (2024): 114750, 10.1016/j.measurement.2024.114750.

[81] N. J. Trujillo , M. C. Barr , S. G. Im , and K. K. Gleason , “Oxidative Chemical Vapor Deposition (oCVD) of Patterned and Functional Grafted Conducting Polymer Nanostructures,” Journal of Materials Chemistry 20, no. 19 (2010): 3968–3972, 10.1039/B925736E.

[82] X. Li , A. Rafie , Y. Y. Smolin , S. Simotwo , V. Kalra , and K. K. S. Lau , “Engineering Conformal Nanoporous Polyaniline via Oxidative Chemical Vapor Deposition and Its Potential Application in Supercapacitors,” Chemical Engineering Science 194 (2019): 156–164, 10.1016/j.ces.2018.06.053.

[83] G. Wang , C. Lu , T. Sun , and Y. Li , “Accelerating the Stabilization of Polyacrylonitrile Fibers by Nitrogen Pretreatment,” Journal of Applied Polymer Science 139, no. 19 (2022): 52129, 10.1002/app.52129.

[84] K. K. Gleason , “Three‐Dimensional (3D) Device Architectures Enabled by Oxidative Chemical Vapor Deposition (oCVD),” Organic Materials 4, no. 04 (2022): 261–267, 10.1055/a-1982-7432.

[85] S. M. Rumrill , V. Agarwal , and K. K. S. Lau , “Conformal Growth of Ultrathin Hydrophilic Coatings on Hydrophobic Surfaces Using Initiated Chemical Vapor Deposition,” Langmuir 37, no. 25 (2021): 7751–7759, 10.1021/acs.langmuir.1c00918. 34125556

[86] Z. Jiang , “GIXSGUI: A MATLAB Toolbox for Grazing‐incidence X‐ray Scattering Data Visualization and Reduction, and Indexing of Buried Three‐dimensional Periodic Nanostructured Films,” Journal of Applied Crystallography 48, no. 3 (2015): 917–926, 10.1107/S1600576715004434.

