# Supplementary material for: Flexible Polypyrrole‐Based pH Sensors via Oxidative Chemical Vapor Deposition
Source: Adv Healthc Mater. 2026 Feb 3;15(16):e05807. doi: 10.1002/adhm.202505807 (PMC13107506; doi:10.1002/adhm.202505807)
Supplement: Supplementary file 1 — Supporting File: adhm70891‐sup‐0001‐SuppMat.docx. [file ADHM-15-0-s001.docx]

**Supporting Information**

Flexible Polypyrrole-based pH Sensors via Oxidative Chemical Vapor Deposition

*Adrivit Mukherjee^a,b,c^, Federico Ferrari^d^, David Garcia Romero^d^, Ilaria Squillante^e^, Job Schoenmaker^a^, Hamoon Hemmatpour^f^, Anton Terpstra^a^, Peter Dijkstra^b^, Julien Es Sayed^b^, L. Jan Anton Koster^d^, Maria Antonietta Loi^d^, Petra Rudolf^f^, Giuseppe Portale^e^, Ajay Giri Prakash Kottapalli^c^, Marleen Kamperman^b^, Ranjita K. Bose^a^**

^a^Chemical Product Engineering, Faculty of Science and Engineering, Engineering and Technology Institute Groningen (ENTEG), University of Groningen, Nijenborgh 3, 9747 AG, Groningen, The Netherlands

^b^Polymer Science, Faculty of Science and Engineering, Zernike Institute for Advanced Materials (ZIAM), University of Groningen, Nijenborgh 3, 9747 AG, Groningen, The Netherlands

^c^Bioinspired MEMS and Biomedical Devices Group, Faculty of Science and Engineering, Engineering and Technology Institute Groningen (ENTEG), University of Groningen, Nijenborgh 4, 9747 AG, Groningen, The Netherlands

^d^Photophysics and Optoelectronics, Faculty of Science and Engineering, Zernike Institute for Advanced Materials (ZIAM), University of Groningen, Nijenborgh 3, 9747 AG, Groningen, The Netherlands

^e^Physical Chemistry of Polymeric and Nanostructured Materials, Faculty of Science and Engineering, Zernike Institute for Advanced Materials (ZIAM), University of Groningen, Nijenborgh 3, 9747 AG, The Netherlands

^f^Surfaces and Thin Films, Faculty of Science and Engineering, Zernike Institute for Advanced Materials (ZIAM), University of Groningen, Nijenborgh 3, 9747 AG, The Netherlands

Corresponding Author

^※*^Ranjita K. Bose – Chemical Product Engineering, Faculty of Science and Engineering, Engineering and Technology Institute Groningen (ENTEG), University of Groningen, Nijenborgh 3, 9747 AG, Groningen, The Netherlands. Email: r.k.bose@rug.nl

**
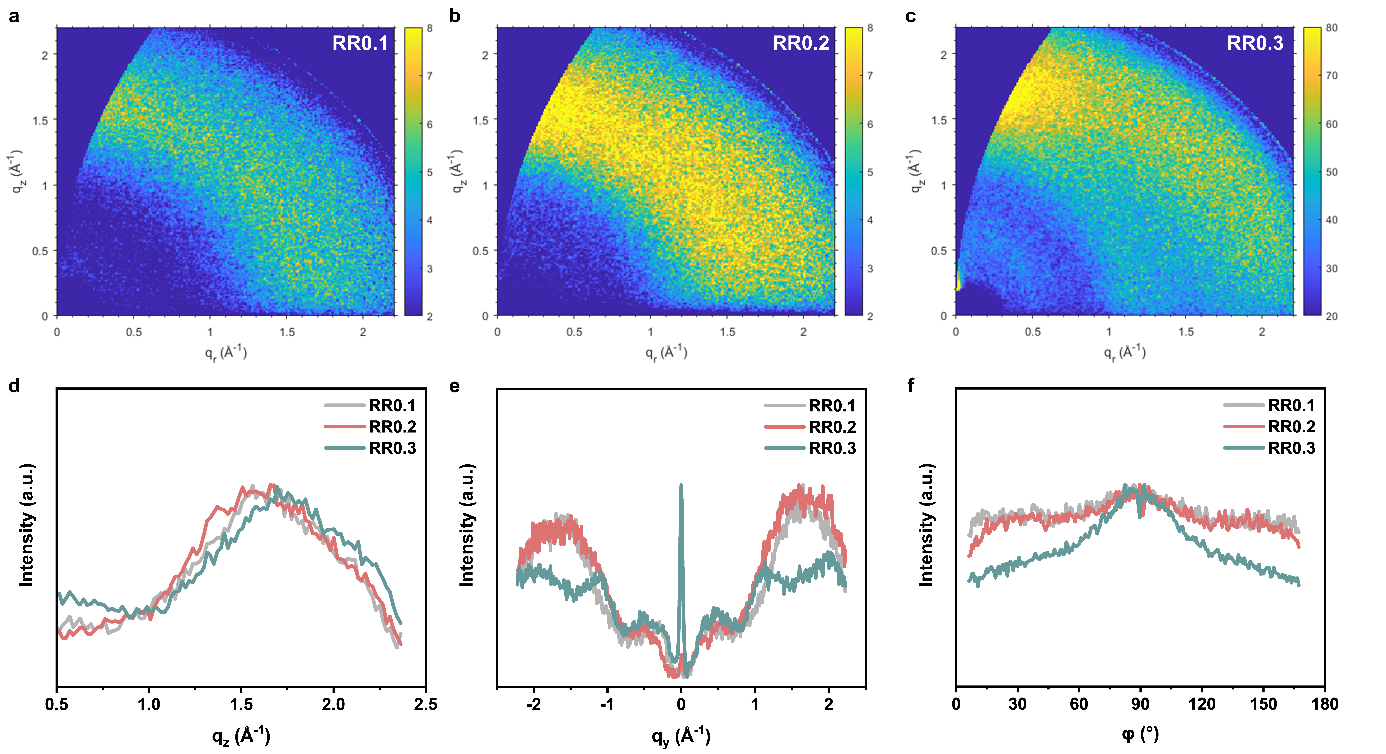
**

Figure S1. 2D GIWAXS patterns of oCVD PPy films with a. RR0.1, b. RR0.2, c. RR0.3. d. Vertical, e. horizontal line cut profiles, and f. azimuthal intensity profile of GIWAXS patterns of oCVD PPy films with varying RR(0.1 – 0.3).


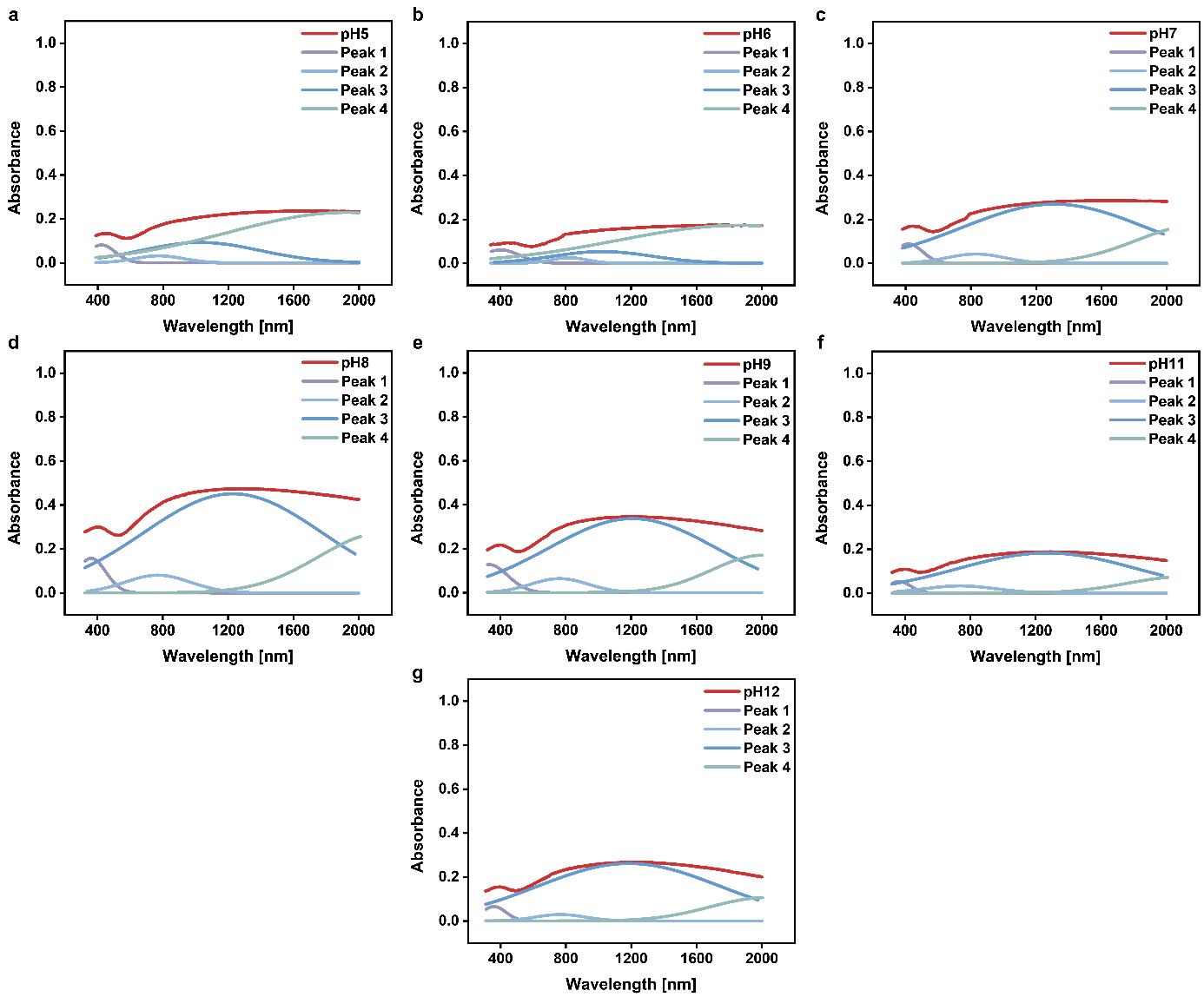


Figure S2. Deconvoluted UV-Vis-NIR spectra of oCVD PPy films equilibrated in solutions of pH ranging between a. 5, b. 6, c. 7, d. 8, e. 9, f. 11, and g. 12, showing the corresponding relative contributions of the neutral, polaron, and bipolaron transitions.


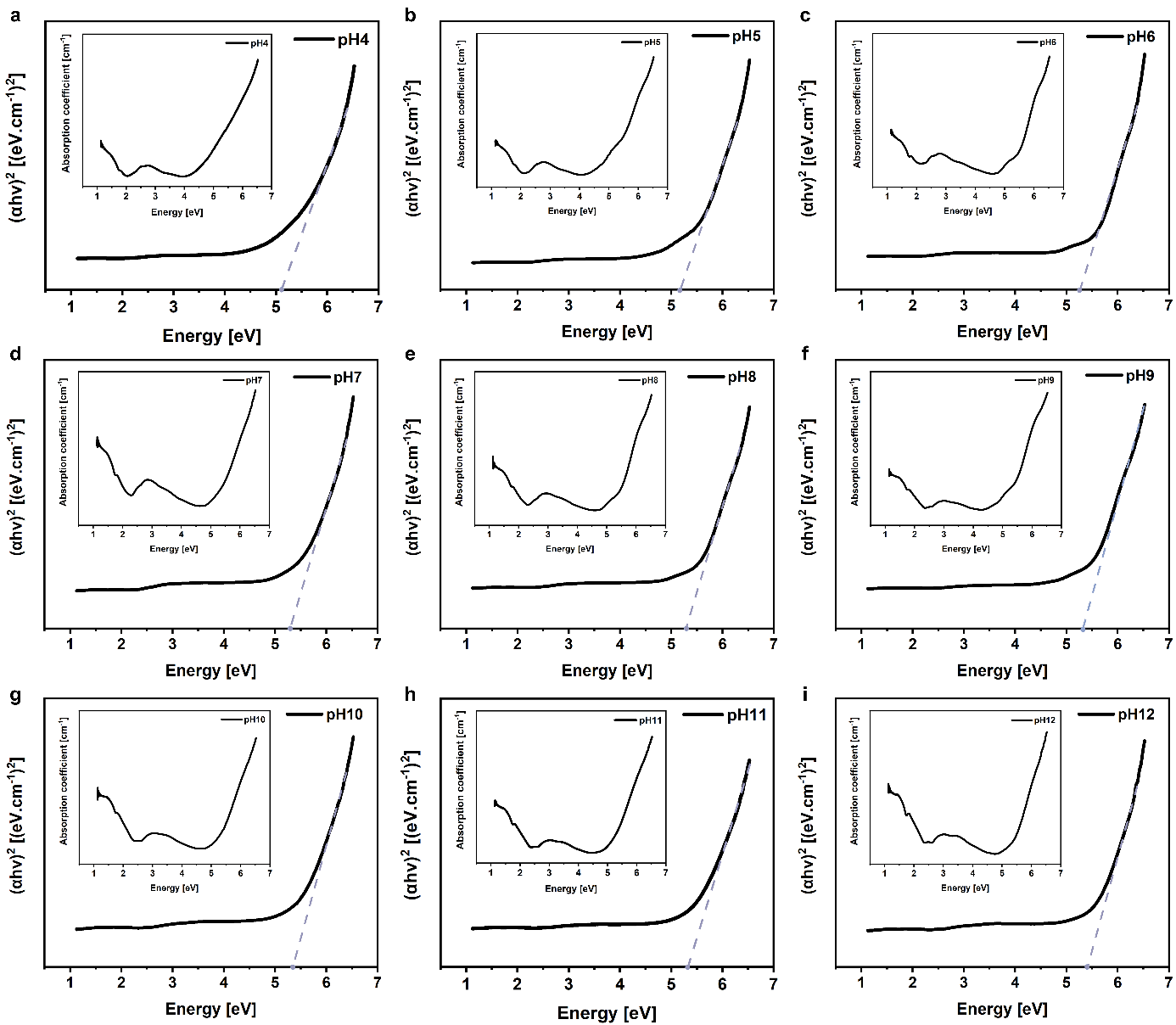


Figure S3. Tauc plots and absorption coefficients (*inset*) for oCVD PPy films equilibrated in solutions of pH a. 4, b. 5, c. 6, d. 7, e. 8, f. 9, g. 10, h. 11, and i. 12, showing the corresponding optical band gap energies.


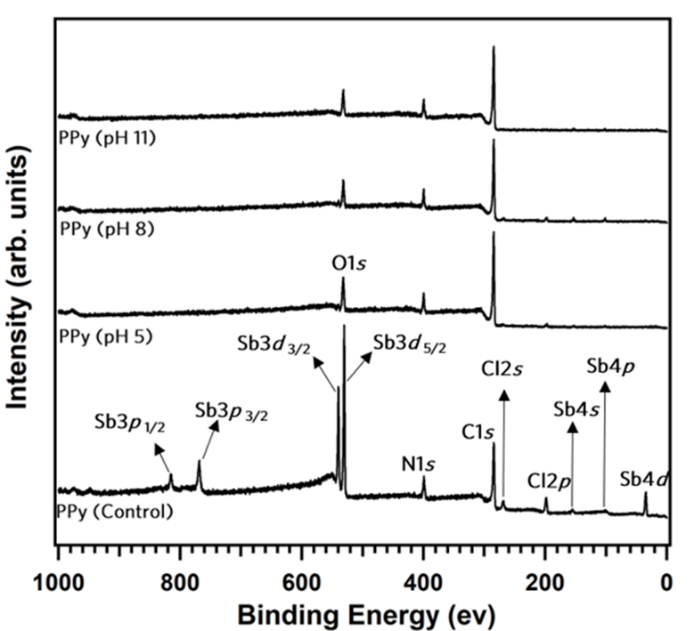


Figure S4. XPS survey spectra for oCVD PPy coatings after exposure to aqueous solutions with different pH levels.


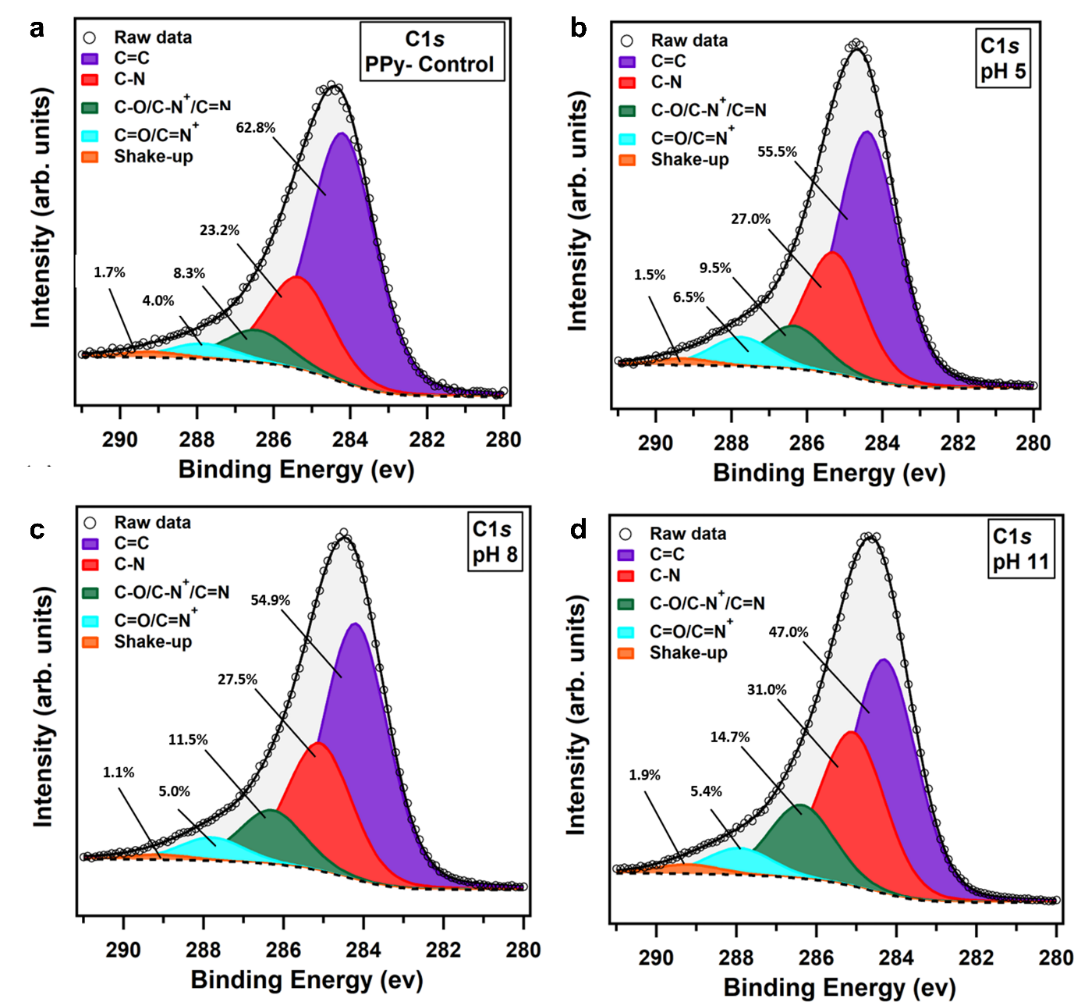


Figure S5. XPS spectra of the C1*s* core level region of PPy coatings after exposure to aqueous solutions with different pH levels.


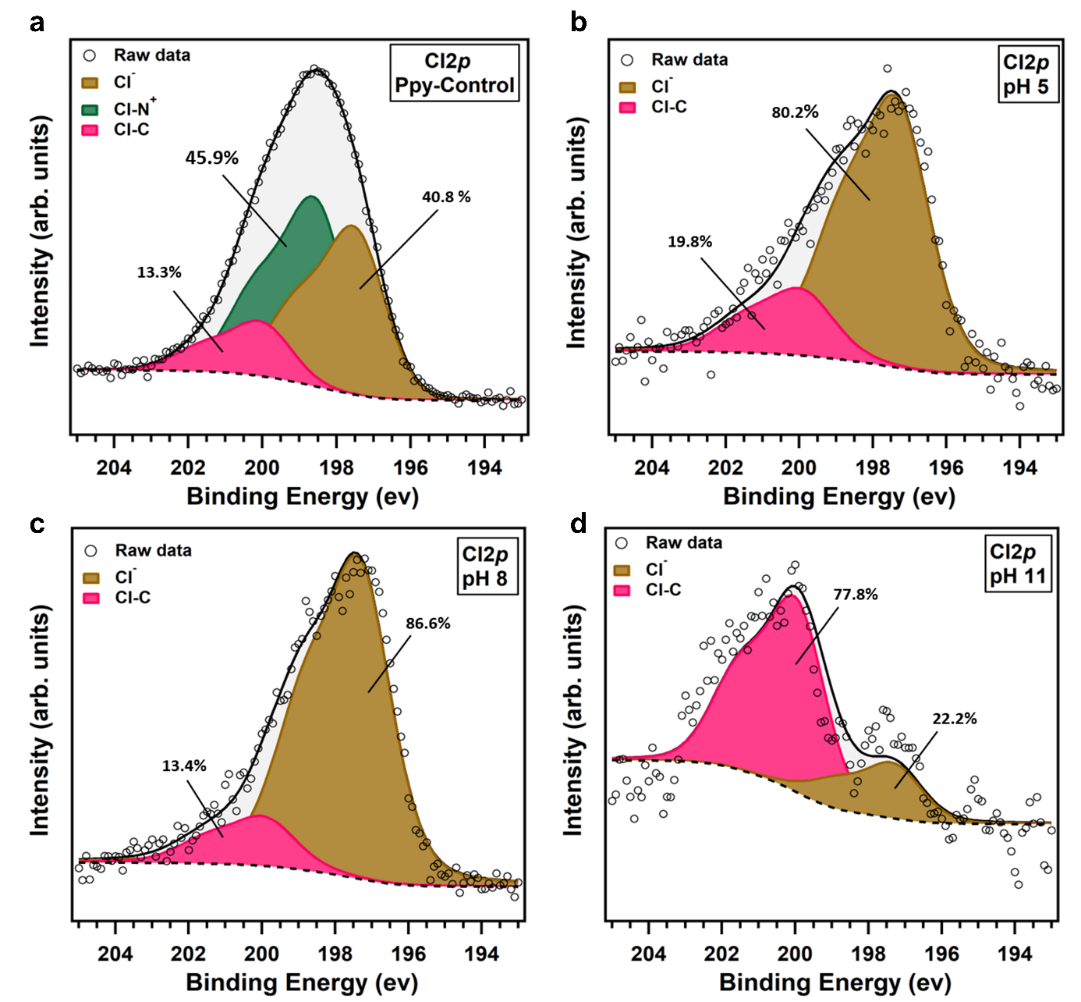


Figure S6. XPS spectra of the Cl2*p* core level region of PPy coatings after exposure to aqueous solutions with different pH levels.


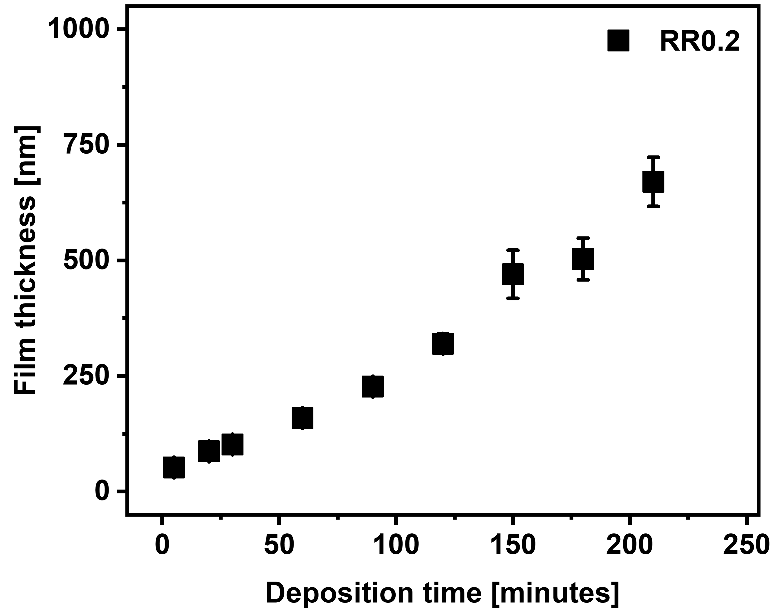


Figure S7. Deposition rate calibration curve of oCVD PPy (RR0.2).


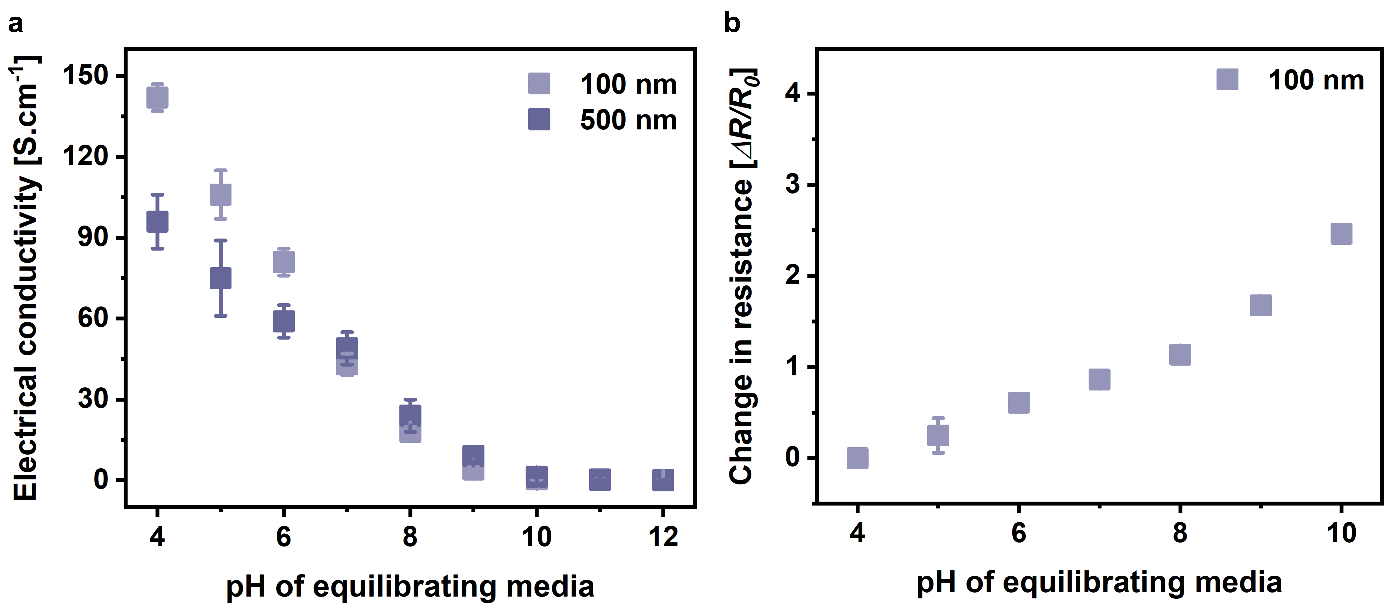


Figure S8a. Electrical conductivity of thin (100 nm) and thick (500 nm) oCVD PPy films as a function of pH of equilibrating media, b. Calibration curve of the relative change in resistance of the pH sensor strip coated with oCVD PPy (100 nm) with increasing pH.


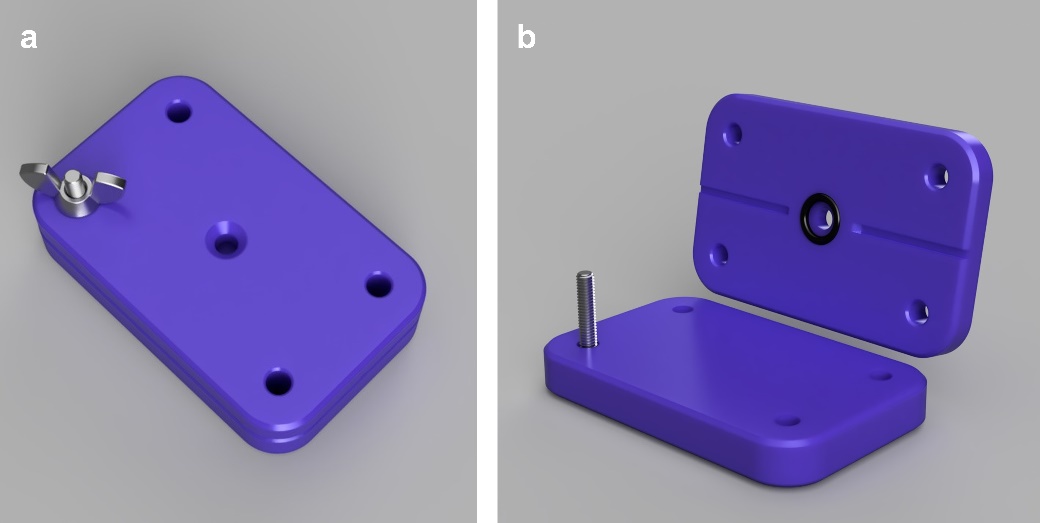


Figure S9. Schematic representation of the custom-built cell used for evaluating the performance of the oCVD PPy-coated pH sensor strip.


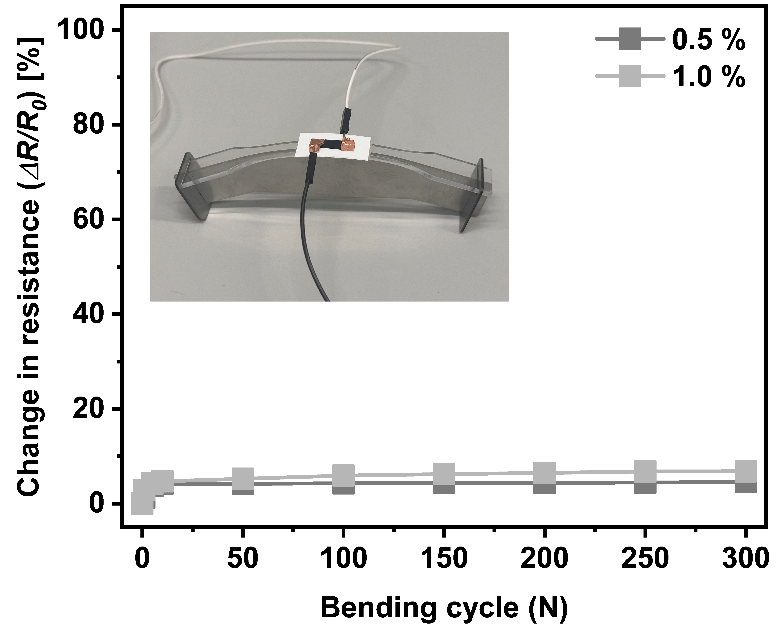


Figure S10. Relative change in resistance of oCVD PPy sensor in cyclic bending tests (0.5% and 1.0% bending strain) over 300 cycles.


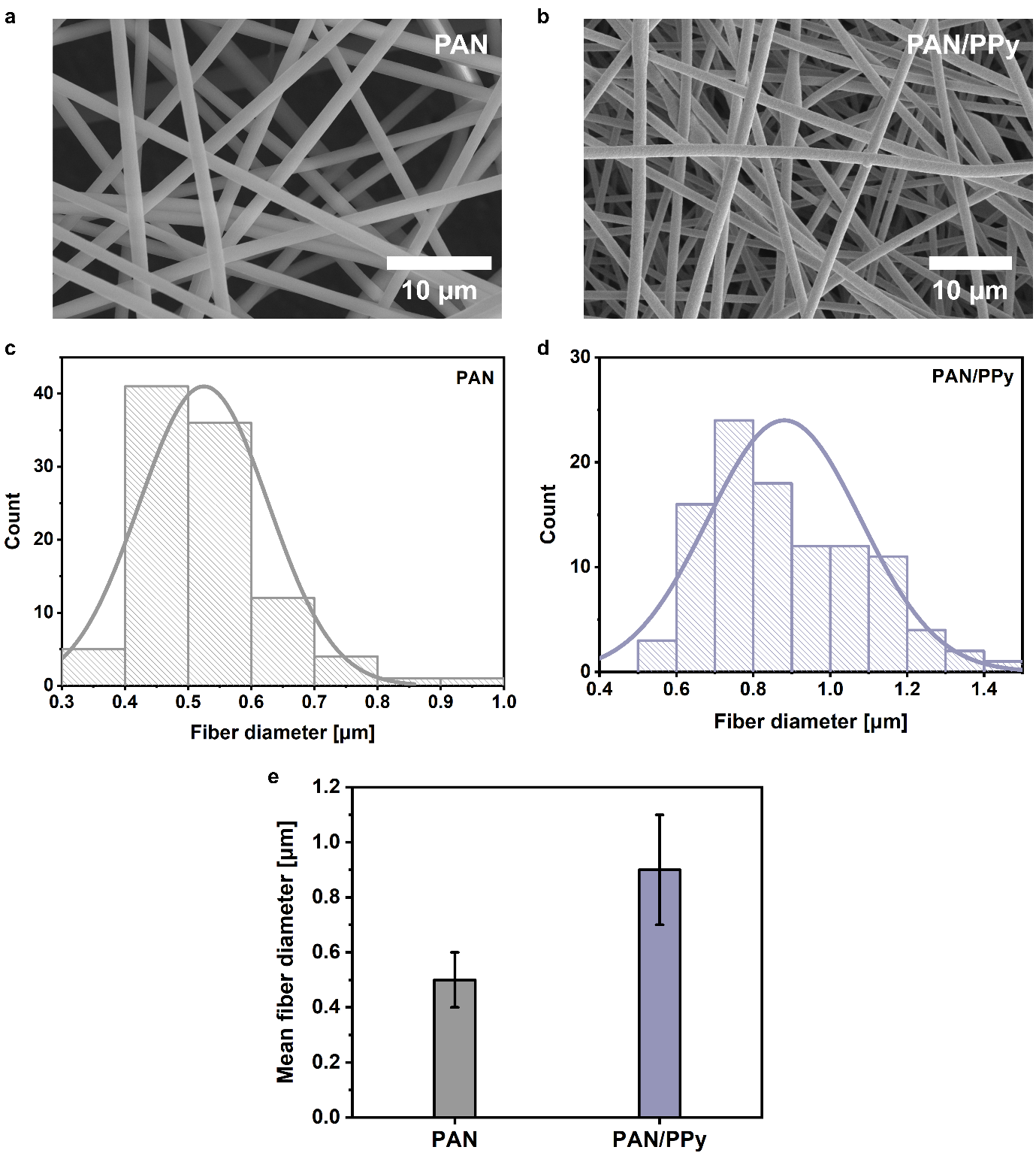


Figure S11. Microstructure of the electrospun fiber mats imaged using SEM, and fiber diameter analysis of pristine PAN (a,c) and PAN/PPy (b,d). Quantitative analysis reveals an increase in the mean fiber diameter after oCVD PPy coating (thickness ≈ 400 nm), indicating successful deposition. Error bars depict standard deviation (*n* = 100).


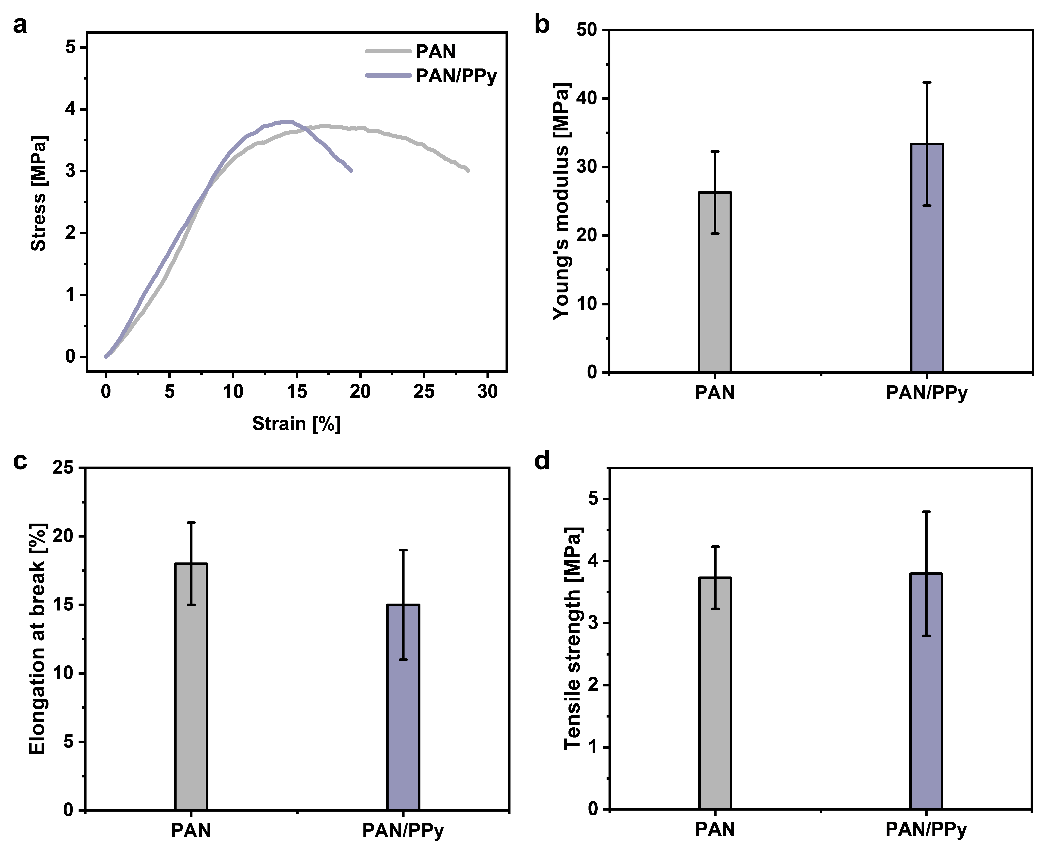


Figure S12a. Uniaxial tensile testing and comparison of the b. Young’s modulus, c. elongation at break, and d. tensile strength of PAN and oCVD PPy coated PAN electrospun fiber mats. Error bars depict standard deviation (*n* = 3).


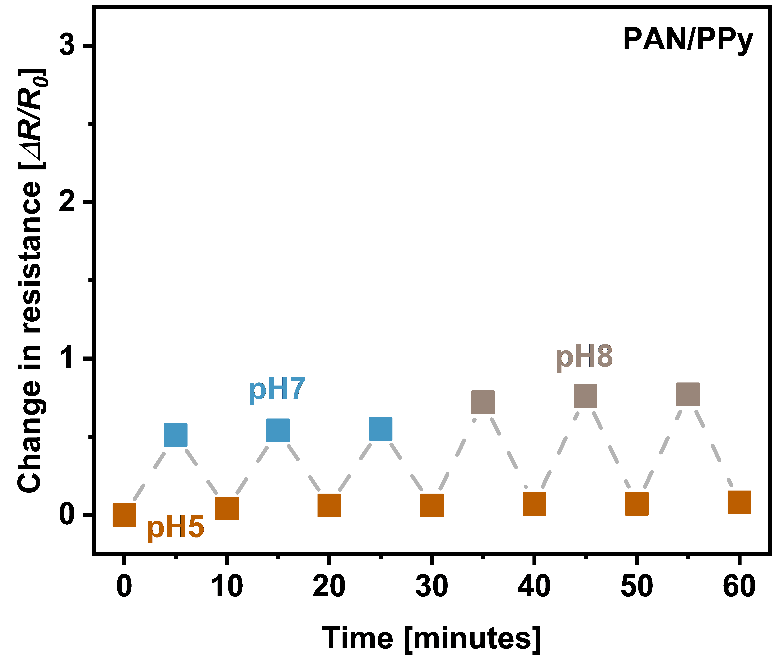


Figure S13. Relative change in resistance of oCVD PPy/PAN electrospun fiber mats during successive dynamic pH cycles (pH 5-7 and pH 5-8).
